# ENTRY IN THE ADHD DRUGS MARKET: WELFARE IMPACT OF GENERICS AND ME-TOO'S*

**DOI:** 10.1111/joie.12017

**Published:** 2013-07-11

**Authors:** Farasat A S Bokhari, Gary M Fournier

**Affiliations:** † School of Economics and Centre for Competition Policy, University of East AngliaNorwich, Norfolk, NR4 7TJ, England; ‡ Department of Economics, Florida State UniversityTallahassee, Florida, 32306, U.S.A

## Abstract

Recent decades have seen a growth in treatments for attention deficit hyperactivity disorder (ADHD) including many branded and generic drugs. In the early 2000's, new drug entry dramatically altered market shares. We estimate a demand system for ADHD drugs and assess the welfare impact of new drugs. We find that entry induced large welfare gains by reducing prices of substitute drugs, and by providing alternative delivery mechanisms for existing molecules. Our results suggest that the success of follow-on patented drugs may come from unanticipated innovations like delivery mechanisms, a factor ignored by proposals to retard new follow-on drug approvals.

## I. Introduction

The hatch-waxman act of 1984 aims to balance the dual objectives of preserving the incentives for undertaking R&D by innovators while at the same time offering incentives for generic entry. Notably, under **S**ection IV of the Act the first successful generic entrant to challenge the patent is granted six months of generic exclusivity (Grabowski and Vernon, [1992, 1996], Grabowski, Vernon and DiMasi [2002], Frank and Salkever [1997], Shulman, DiMasi and Kaitin, [1999]). In recent years, actions by pharmaceutical firms (as well as approval policies at the Food and Drug Agency (FDA)) have come under scrutiny for potentially undermining the intent of the Act. For instance, the introduction of follow-on drugs (the so called me-too's) is criticized because they reduce the profits of the innovator and hence the incentives to engage in R&D, without necessarily offering either price reductions or significant therapeutic benefits to consumers.^1^ Similarly, the entry of an authorized generic drug under a license from the innovator raises concerns since it discourages other generic drug firms from pursuing entry. In terms of consumer welfare, the latter issue is further complicated because the licensed generic entry often takes place well before the patent expiration of the innovator, but perhaps later than it would have otherwise occurred under the section IV terms, as suggested by ‘reverse payments’ made by the patent holder to the licensee (Bulow [2004], Reiffen and Ward [2007], Berndt, Mortimer, Bhattacharjya *et* *al*. [2007], Frank [2007], FTC [2010]).

The introduction of new products expands the range of consumer choice and increases consumer welfare. The magnitude of welfare effects in turn depends partly on the level of product differentiation, the steepness of the individual demand curves and cross-elasticities of demand, as well as the induced effects on price competition among incumbents and new entrants (Bresnahan [1997]). These interactions play an especially prominent role in the pharmaceutical industry where even modest differentiation may lead to large welfare gains. Consider the introduction of a generic drug. While a generic does not introduce a new product variety, it may still create large welfare gains if the market expands to include price sensitive consumers who formerly were either consuming a drug in a different molecule class, a different form, or doing without drug therapy. The welfare analysis of generic entry becomes somewhat more complicated when we recognize the possibility of price *increases* for branded drugs in the brand loyal segment of the market as a response to generic entry (Grabowski and Vernon [1992], Frank and Salkever [1992], Regan [2008]).

The magnitude of welfare effects of the me-too drugs is also ambiguous. On one hand, Lu and Comanor [1998] report that in the U.S., me-too drugs were typically introduced at the same price as the original branded drugs, and the average effect of adding an extra competitor was a price reduction of about 2%. Similarly, Lichtenberg and Philipson [2002] report that ‘between-patent’ competition may reduce an innovator's returns at least as much as that from ‘within-patent’ competition (the term ‘between-patent’ refers to competition from other drugs in the class and loosely corresponds to the me-too's while the latter refers to competition from generics). On the other hand, DiMasi and Paquette [2004] suggest that me-too drugs may provide substantial welfare gains by lowering side effects, changing the delivery mechanism, or targeting a new sub-population and effectively increasing the market.

In this paper we estimate a demand system for psychostimulant drugs—a segment fraught with the issues mentioned above—and use our estimates to gauge the potential welfare gains due to the introduction of generics as well as of me-too's. We also discuss the likely welfare loss due to the delayed entry of a generic in this market. The demand for psychostimulant drugs used to treat Attention Deficit Hyperactivity Disorder (ADHD) has grown rapidly in the past decade. Between 1990 and 1996, psychostimulant consumption increased 37% nationwide, while the number of patients diagnosed with the disorder grew from around 900,000 to approximately 3 million. In 2000, the total sales of ADHD drugs in the U.S. were about $1 billion, and by 2003 had surpassed $2.2 billion (in constant 2000 dollars). This explosion in the market allowed several drug manufacturers to enter the ADHD market. By the late 1990's, there were at least half a dozen different branded drugs in this market (some were still on-patent) as well as many generic equivalents of expired patent formulas. The entry by new drugs has evolved into a large differentiated product system containing both branded and generic drugs. These new drugs were either new entities (i.e., new formulas or molecules) or new presentations (i.e., new forms that extend the release) and were introduced by incumbent drug firms as well as by new entrants.

Some of the new introductions were almost overnight successes. Concerta was introduced in 2000 and immediately secured 4.7% of the market and by 2003 was a ‘blockbuster’ with a market share of 26.1% of all ADHD drugs. Another blockbuster, Adderall XR, was introduced in 2001 by the incumbent firm Shire which had been marketing Adderall since 1996. Both Adderall and Adderall XR are mixed amphetamine salt based molecules (MAS) targeted for populations for whom the traditional methylphenidate molecule (MPH) may not be as effective, and where XR is the extended release version (MAS-ER) while Adderall is the immediate release version (MAS-IR). In 2001, the market share of Adderall was 35.8% and that of Adderall XR was 1.1%. However, by 2003 the share of Adderall was 2.9% while that of Adderall XR was 23.8%. While this may be a case of a firm's ‘cannibalizing’ its own product (and shifting market shares), it can be argued that without such a move, Shire would have lost significant market share to the generic entry in the MAS-IR segment that took place in 2002. Additionally, Shire also faced a threat of entry for its Adderall XR product when a generic manufacturer (Barr laboratories) filed for an Abbreviated New Drug Application (ANDA) with the FDA in February, 2003. Shire sued for infringement of its key patents on the Adderall XR and eventually Shire and Barr reached an out of court settlement. Under the terms of the agreement, Barr agreed not to enter until April, 2009, at which point it would enter as a licensed generic maker of Adderall XR with a 180-day exclusivity period.

Following Hausman, Leonard and Zona [1994] and Ellison, Cockburn, Griliches *et* *al*. [1997], we use the assumption of weak separability and multistage budgeting by a representative consumer to divide the market into smaller segments in a nested demand system. Our nesting structure is based on pharmacological differences among various drugs and how they segment the market (and is described in the next section). The lowest segments consist of individual drugs within the same molecule and form. The next level up consists of different forms of the drug in the same molecule. Level 3 consists of choice across molecules, and finally at the top-level we estimate a single demand equation which consists of all psychostimulant drugs used for the treatment of ADHD. Section 3 describes the data and section 4 lays out the empirical specification. Since prices are endogenously determined, we rely on the common cost shocks identification strategy used by Hausman [1997] to instrument for the price of a drug in a given market by its price from another geographic market. Results and welfare calculations are given in section 5. The last section concludes.

## II. Growth and Product Differentiation

### II(i). Market Expansion

The demand for drugs to treat ADHD has grown rapidly in the past decade. It is the most commonly diagnosed behavioral disorder in children and approximately 3–5% of school-age children have this disorder; some estimates range as high as 7–12% or between 1.5–6 million children. About 75–80% of children diagnosed with ADHD are treated with psychostimulant drugs. Rates of psychostimulant drug use vary as much as 3-fold between states and 10-fold within them (Cantwell [1996], Zito, Safer, Riddle *et* *al*. [1998], Lefever, Dawson and Morrow [1999], Cox, Motheral, Henderson *et* *al*. [2003]).

Sales of several psychostimulant drugs can be traced back to at least the 1950's. These drugs include some that were specifically approved by the FDA to treat behavioral disorders, as well as off-label drugs that were federally approved for other purposes yet were routinely prescribed by physicians for the treatment of ADHD (ADHD was officially recognized as a disorder by the National Institute of Mental Health (NIMH) in 1980). For instance, methylphenidate-HCL (MPH) patented in 1954 by Ciba Pharmaceutical, was marketed under the trade name of Ritalin for the treatment of chronic fatigue, depression and narcolepsy as well as to offset the sedating effects of other medications.^2^ The FDA approved methylphenidate for the treatment of ‘functional behavior problems’ in 1963, and by 1966, Ritalin was often recommended for children with ‘Minimal Brain Dysfunction (MBD)’. Sales of methylphenidates grew steadily over the 1970's and 1980's and got a big boost in the early 1990's after the publication of studies showing marked improvement in the school performance of children suffering from ADHD and on drug therapy (see Evans and Pelham [1991] and Carlson, Pelham, Milich *et* *al*. [1992]). Over the same period, other molecules had gained acceptance for treating ADHD. For instance, Obetrol, which consists of four mixed dextro and levoamphetamine salts, had been unsuccessfully on the market since the 1960's as an approved obesity drug. In 1994, the rights to the Obetrol formulation were sold to Rexar, which was subsequently acquired by Shire. In turn, Shire received approval from the FDA in 1996 to market the mixed amphetamine salts (MAS) formulation to treat ADHD and sold it under the brand name Adderall.

Significant growth in psychostimulant drug use began in the early 1990's soon after major changes were enacted by policymakers in Washington, D.C., to include ADHD as a protected disability under the Supplemental Security Income (SSI) program and the Individuals With Disabilities Education Act (IDEA) (Safer, Zito and Fine [1996], Zito, Safer, dosReis *et* *al*. [2000], Bokhari, Mayes and Scheffler [2005]). The tightening of school accountability laws over time also contributed to increased diagnosis of ADHD and demand for psychostimulant drugs (Bokhari and Schneider [2011]). Concurrently, several state Medicaid programs ‘carved out’ their mental health benefits to speciality firms during the 1990's, which also led to an increase in demand for various psychotropic drugs (Ling, Berndt and Frank [2008]). This expansion in the market allowed for several drug manufacturers to enter the ADHD market. Over the same period, promotional activities (physician detailing, journal advertising, free samples, direct to consumer advertising) for prescription drugs also increased dramatically and may have further contributed to an increased demand for ADHD drugs (total spending on promotions grew at an average annual rate of 10.6% between 1996 and 2005 (Donohue, Cevasco and Rosenthal [2007])). In September, 2001, pharmaceutical companies that produced ADHD drugs broke a 30-year agreement with the Drug Enforcement Agency (DEA) and the FDA not to advertise their Schedule II controlled substances directly to consumers. As a percentage of sales, the three leading brands, Adderall XR, Concerta and Strattera (a non-stimulant) spent 6.75%, 4.2% and 16.7% on direct-to-consumer advertising in 2003.

### II(ii). Product Differentiation—Role of Molecules and Forms

ADHD is a behavioral disorder marked by excessive inattentiveness and/or hyperactivity-impulsivity. Children with ADHD are believed to have abnormal functioning, or dysregulation, of certain brain chemicals known as neurotransmitters (chemical messengers). ADHD drugs boost levels of two such neurotransmitters, dopamine and norepinephrine, which help to regulate attention and activity. Dopamine is thought to play a role in memory formation and the onset of addictive behaviors, while norepinephrine has been linked with arousal and attentiveness. ADHD drugs increase the levels of norepinephrine and dopamine by either inhibiting their reabsorption (reuptake) into cells or by promoting the release of these chemicals from the brain. For instance, methylphenidate based ADHD drugs, such as Ritalin, inhibit the reuptake of dopamine into cells, whereas amphetamine based drugs, such as Adderall, while inhibiting the reuptake of dopamine, also promote its release into the brain. Depending on the physiology of a patient, one molecule may be more effective than another. Additionally, a particular molecule in a given person may induce adverse reactions. Physicians and patients often have to experiment with different molecules to help identify which one is most suitable for a given patient (or rule out those that induce adverse reactions).

Once a molecule is selected, several delivery mechanisms are available which can significantly affect the choice of a specific drug. The primary differences are in the absorption rate into the blood stream and the time to peak effect. Drugs are available in immediate-release (IR) tablets or liquid form as well as in extended-release (ER) tablets or capsules. Immediate release formulas, such as Ritalin or Adderall, typically last three to four hours and are taken two or three times a day. These formulations can be more tightly controlled in terms of dosage and frequency in order to inhibit the reuptake and/or promote additional release of neurotransmitters. In the extended release formulations, part of the drug is released immediately into the blood stream while the remaining drug in the capsule is released more slowly and at different rates. These are often further differentiated into intermediate-acting extended-release tablets, such as Ritalin LA or Metadate CD that may last six to eight hours, or long-acting extended-release capsules and tablets such as Concerta that last eight to twelve hours.^3^ The extended release forms reduce the peaks and troughs (‘ups and downs’) over the day and eliminate the need for additional doses during school hours. Thus, each delivery mechanism comes with its own advantages and disadvantages and further segments the market into subgroups (for an accessible reading see Barkley [2006], Conner [2006] and Spencer [2006]—chapters 5, 17 and 18—in the Barkley [2006] handbook on ADHD diagnosis and treatment).

Table I lists drugs by groups that that are deemed medically similar by health care professionals such that those within the same group can be substituted gram for gram, while those in different subgroups require dosing adjustments.^4^ This is not to say that drugs in the same group are always generic equivalents of each other. For instance, while Ritalin LA and Metadate CD are in the same group, they embody slightly different delivery mechanisms (see footnote (3)). The table also provides an approximate rule that physicians employ when switching a patient's drug across a group. The switch from Concerta to Ritalin requires a dosing adjustment such that if a child was previously consuming 1 mg of Concerta over a period of time, they would now use only 0.69 mg of Ritalin over the same period.

**Table I:** Groups of Bio-equivalent Drugs

*Methylphenidates* (MPH)			
IR ∼ 4hr (1.0)	ER-TAB ∼ 8hr (.83)	ER-CAP ∼ 8hr (1.25)	OROS ∼ 12hr (0.69)
Ritalin	Ritalin SR	Ritalin LA	Concerta
Methylin	Methylin SR	Metadate CD	
Generic Ritalin	Metadate ER		
	Generic Ritalin SR		
*Mixed Amphetamine Salts* (MAS)			
IR ∼ 4hr (2.86)	ER ∼ 12hr (2.14)		
Adderall	Adderall XR		
Generic Adderall			
*Dextroamphetamines* (DEX)			
IR ∼ 4hr (1.75)	ER ∼ 8hr (2.14)		
Dexedrine	Dexedrine SR		
Dextrostat	Generic Dexedrine SR		
Generic Dexedrine			
*Other molecules* (OTH)			
(.28)	(.44)	(.83)	
Provigil	Cylert	Strattera	
(Modafinil)	(Pemoline)	(Atomoxetine)	
	Generic Pemoline		

The key feature of this table, and one that informs our estimation strategy, is that drugs that can be substituted gram for gram have the same molecule and form. For instance, within the methylphenidate based drugs, there are four subgroups by dosage equivalence. Further, these subgroups differ precisely by the delivery mechanisms mentioned earlier: immediate release (IR), intermediate-acting extended-release (ER-TAB), long-acting extended-release (ER-CAP) and Concerta which is in a category by itself.

## III. Data

### III(i). Data Source

Data for this study were obtained from the NDCHealth's proprietary Source Territory Manager® data files for the years 1999–2003. NDCHealth's data set provides at the retail level, total sales (in dollars) and number of pills dispensed by strength (in milligrams) for several branded and generic versions of ADHD related drugs at the 5-digit ZIP code level. The Source Territory Manager's coverage is about 70% of all retail level sales (the remaining 30% are pharmacies typically from rural areas). Thus, for each 5-digit ZIP code in the coverage area and for each year, we know, for instance, the number of pills dispensed for each strength of Ritalin (5mg, 10mg and 20mg) as well as the total revenue collected by the retailer from all parties (insurance plus co-pay) for each strength separately. Similar information is known for other forms of the drug. Using the number of pills dispensed times strength, we obtain the total grams for each drug and form in the local ZIP code area and then aggregate the quantities and revenues up to county level. Dividing the total revenue by the total grams gives a measure of the average price in the county year for the drug-form. Note two features of this measure of the price: [Disp-formula m1a] [Disp-formula m1b] [Disp-formula m1c] (1) it is not a list price but is closer to the (average) transaction level price, and (2) since it is based on retail level data (rather than wholesale), it incorporates the final price of the product paid by all parties (private or public insurance and out of pocket payments) and not just the co-payment component paid by the consumer. However, it is not exactly equal to the average transaction level price because our sales data do not capture rebates. For instance, state Medicaid programs, which make payments to the pharmacies, receive a rebate from participating manufacturers under the Medicaid Drug Rebate Program. These rebates have not been subtracted out of the sales data prior to dividing by quantities.

### III(ii). Sample

For our analysis, we restricted the sample to counties within all Metropolitan Statistical Areas (MSA's), i.e., to 852 counties. This choice is dictated by two factors. First, not all drugs are necessarily consumed in a given county-year, especially in rural counties. Thus, while the quantity (or share) is known to be zero, the price is not known since it is derived as the ratio of sales to quantity. Including these counties would necessitate imputing the price. However, the problem is largely avoided if we restrict the sample to MSA counties. Second, rural counties also have very few physicians, and since the choice of a drug is in part due to a physician's experience with a specific brand, the demand parameters for rural areas may be very different from those in urban areas. Mixing the two populations may provide an average effect of price on demand, but may not in fact be representative of demand for either the rural or urban populations. Thus, we chose to restrict our analysis to counties in MSA's and imputed the price of a drug as equal to the state year average if sales in the county were zero. Finally, we further restricted the sample to counties with ‘balanced’ observations across years, i.e., if the drug is on the market, we must be able to observe (positive) sales for all the years since introduction. This criteria reduced the working sample further to 778 counties.

For practical reasons, we have also omitted two drugs from our analysis. The first is Desoxyn which is a methamphetamine molecule. It is legally produced only by Ovation Pharmaceuticals, however the drug is also available illegally through its production in clandestine laboratories throughout the United States, and goes by the street name ‘ice.’ The data from legal sales was sparse (less than .15% of sales) but it generally sells for more than $200 per gram. The second drug omitted from our analysis is Focalin which is a close cousin of the MPH molecule except that it is a single isomer of MPH. It was introduced in 2002 by Novartis but never attained more than .5% of the market share during the observation period. We cannot estimate the price of this drug reliably since it was sold in very few areas. Thus, in our representative consumer model, these two drugs can be thought of as belonging to the group, ‘all other goods,’ since both consist of molecules different from those considered in this study. For the remaining drugs, price per gram and shares are summarized by year in Table II (all dollar figures throughout the paper are expressed in constant 2000 dollars and were deflated using the CPI).

**Table II:** Average Prices and Shares by Year (778 MSA Counties)

		Avg. Price Per Grm/Std					Avg. Share of Revenue				
		1999	2000	2001	2002	2003	1999	2000	2001	2002	2003
(1)	Ritalin (1.0)	54/3.9	53.32/3.8	52.81/4.4	53.61/5	58.23/6.5	0.117	0.081	0.033	0.016	0.009
(2)	Methylin (1.0)	45.26/6	41.87/5.5	39.95/6.6	37.92/6.6	35.34/7	0.045	0.068	0.035	0.022	0.013
(3)	Gen MPH-IR (1.0)	43.69/3	41.55/4.2	39.74/4.3	38.58/4.4	36.73/4.5	0.289	0.185	0.098	0.049	0.026
(4a)	Ritalin SR (0.83)	61.26/2.1	61.38/2.2	62.34/2.4	64.4/3.2	70.49/3.7	0.047	0.032	0.012	0.006	0.003
(4b)	Ritalin LA (1.25)	.	.	.	79.08/8.7	79.51/6.4	.	.	.	0.006	0.024
(5a)	Metadate CD (1.25)	.	.	53.3/2.2	59.8/2.4	78.09/3.4	.	.	0.006	0.025	0.024
(5b)	Metadate ER (0.83)	NA	60.25/12.3	59.28/9.5	59.71/9.7	60.97/11.1	NA	0.007	0.007	0.005	0.002
(6)	Methylin ER (0.83)	.	53.87/9.4	52.22/8.8	48.51/7.3	48.22/7.1	.	0.004	0.010	0.008	0.006
(7)	Gen MPH-ER (0.83)	49.99/2.5	48.68/3.2	46.37/4.9	46.08/5.1	44.38/5.8	0.105	0.080	0.034	0.017	0.008
(8)	Concerta (0.69)	.	84.58/7.6	71.27/5.8	69.63/4.6	73.94/4.3	.	0.047	0.238	0.298	0.261
(9)	Adderall (2.86)	56.53/2.5	63.12/2.9	93.68/8.8	101.7/12.5	101.28/16.9	0.216	0.311	0.358	0.114	0.029
(10)	Gen MAS-IR (2.86)	.	.	.	92.22/13.4	84.29/13.1	.	.	.	0.084	0.076
(11)	Adderall XR (2.14)	.	.	112.8/15.8	116.85/9.2	125.02/8.8	.	.	0.011	0.202	0.238
(12)	Dexedrine (1.75)	49.68/1.9	53.2/2.2	59.13/2.8	66.17/3.6	67.93/3.8	0.013	0.010	0.006	0.003	0.002
(13)	Dextrostat (1.75)	42.06/2.4	45.87/2.7	54.49/1.9	55.99/4.2	45.2/4.2	0.016	0.018	0.012	0.005	0.002
(14)	Gen DEX-IR (1.75)	.	.	51.15/4.6	49.14/4.9	47.87/4.1	.	.	0.003	0.004	0.004
(15)	Dexedrine SR (2.14)	67.64/4.2	76.28/4.8	85.22/5.3	94.15/6.8	95.71/9.6	0.062	0.062	0.045	0.022	0.007
(16)	Gen DEX-ER (2.14)	.	.	.	84.62/7.2	83.55/7.9	.	.	.	0.009	0.011
(17a)	Cylert (0.44)	39.62/2.1	41.99/3	42.6/3.8	44.29/4.6	44.03/4.5	0.061	0.023	0.009	0.004	0.002
(17b)	Provigil(0.28)	24.9/2.3	24.62/1.6	26.11/1.7	26.9/1.6	27.95/1.6	0.022	0.058	0.072	0.093	0.094
(17c)	Gen Pemoline (0.44)	32.47/4	31.66/3.9	33.09/4.5	31.46/5.4	29.57/6.6	0.005	0.015	0.011	0.006	0.004
(17d)	Strattera (0.83)	.	.	.	.	77.09/5.9	.	.	.	0.000	0.156
Total Revenue (in Millions)							688.5	836.8	1,110.3	1,438.0	1,992.7

*Note 1:* Prices are in constant 2000 dollars per gram. A missing value implies the drug was not on the market, except for Metadate ER which was on the market in 1999, but data are not available to us.

*Note 2:* Total revenue (also in constant 2000 dollars) is only for the drugs listed above from the 778 counties and does not include mail order sales.

*Note 3:* The number in parenthesis is the conversion factor used for converting to generic MPH-IR equivalent dosage prices in the main analysis. Example: the price of Concerta per gram in 2003 is $73.94, but 1gm of Concerta is equivalent to 0.69mg of Ritalin and hence the dosage adjusted price of Concerta is $73.94/0.69 = $107.17. This amount can be read as ‘price per defined monthly dosage’.

### III(iii). Descriptive Statistics

In 1999, Ritalin had 11.7% of the market share while its bio-equivalent generic version, immediate release methylphenidate (MPH-IR) had 28.9% of the market share (produced by 15 firms in 2003). New drugs entered the market in 2000 and by 2003, both Ritalin and its generic version had lost significant market share and were down to 0.9% and 2.6% respectively. Over the same period, the average price of Ritalin stayed fairly constant (except for a spike in 2003) while the price of the generics steadily declined.

Concerta entered the market in 2000 and Adderall XR entered in 2001. While both started with modest shares in the year of their introduction, by 2003 these two drugs had achieved nearly 50% of the entire ADHD drug market (26.1% and 23.4% respectively), and sold for $73.94 and $125.02 per gram. Concerta, produced by Ortho-McNeil, introduced its product in a new niche market. Ortho-McNeil entered into an agreement with ALZA, the developers of Concerta, starting in 2000. Concerta itself consists of a time released version of the methylphenidate HCL molecule. However, ALZA developed Concerta by applying Osmotic Release Oral System technology (OROS) for its delivery mechanism. While OROS is also an ER formulation, it is slowly released throughout the day at an increasing rate. Thus, while other extended release formulations of the MPH molecule already existed in the market (eg., Ritalin SR and its generic versions), the OROS technology used by Concerta was the first and only drug to embody a truly new delivery mechanism in any of the ADHD class of drugs. Similarly, until the introduction of Adderall XR, no drug was available in extended release form for the mixed amphetamine salt (MAS) and when Shire introduced this drug, it too created a new niche market. Shire currently holds a patent on the XR version which will expire in 2018.

Another important drug that entered the market is Strattera, a non-stimulant molecule (atomoxetine), introduced in December, 2002, by Eli Lilly. It attained a significant market share in 2003 (about 15%), perhaps because it is the only non-stimulant ADHD drug on the market. Unfortunately, our data series ends in 2003 and hence we will not be able to estimate the individual demand parameters for this drug (in our demand analysis we lump it into a group called ‘other ADHD drugs’ and only estimate the joint effects of this broader category).

The generic version of MAS-IR (i.e., generic Adderall) entered in 2002 and by 2003 had a 7.6% market share (distributed over three firms). Note also that Adderall, the branded drug, enjoyed significant market share up until the introduction of the generic version in 2002: 21.6% in 1999, 35.8% in 2001 and then declined to 11.4% in 2002 when the generic entry took place.

Three other drugs of interest that entered over the study period are Methylin ER, Metadate CD and Ritalin LA. All three are extended release forms of methylphenidate HCL. Methylin ER, introduced in 2000 at $53.76 by Mallinckrodt is about $7 above the average price of other generics and about $7 below the price of Ritalin SR or Metadate ER.^5^ The market share of Methylin ER was .4% while that of Metadate ER and Ritalin SR was .7% and 3.2% respectively. In the following year Celltech, which was already marketing Metadate ER, launched a new time released capsule version, Metadate CD.^6^ This resulted in a total market share of 1.3% (= .6+.7) for Celltech via its two forms of Metadate while the share of Novartis's Ritalin SR declined to 1.2%. In the year following that, Novartis launched it own version of a time released capsule, Ritalin LA.^7^ The introductory price of $79 per gram for Ritalin LA was $20 higher than the pharmacologically closest substitute, Metadate CD. The market share of Novartis stayed at 1.2% (split as .6% and .6% across LA and SR) while the market share of Celltech climbed up to 3% (2.5% for CD and .5% for ER). In 2003, Celltech increased the price of Metadate CD by almost $19 to $78 (which is just $1.4 below that of Ritalin LA in 2003) while its market share declined by .1% down to 2.4%. Ritalin LA gained a significant market share over the previous year from .6% to 2.4%.

Generally, Celltech kept the price of its products Metadate ER and CD slightly below that of the relatively more well known brands Ritalin SR and LA respectively (with some exceptions) and by 2003 had attained a market share of 2.4% which is at par with those of Ritalin SR/LA. On the other hand, Mallinckrodt's Methylin ER was typically priced slightly above that of the generics and attained a .6% market share by 2003, compared to the share of .8% of MPH-ER distributed among 12 generic makers. These are far more modest shares compared to the success of the blockbusters discussed earlier, but still large by industry standards. Further, while such descriptive analysis cannot account for (or hold constant) other simultaneous changes in the market, it appears that Metadate ER/CD are closer substitutes for Ritlan LA/SR while Methylin ER may be a closer substitute for the generic MPH-ER.

## IV. Multistage Budgeting and Conditional Demand Functions

A fundamental problem in estimating a system of demand equations for a set of differentiated products is the problem of dimensionality. For a system with *I* products, the demand system *q* = *D*(*p*, *z*) involves estimation of *I*^2^ parameters, where *p* is the vector of all prices and *z* is the vector of exogenous variables that enter the demand equations. Even if symmetry of the Slutsky matrix, homogeneity and other restrictions are imposed, the number of parameters is still large and increases in the square of the number of products. Depending on the research question at hand, the empirical literature has dealt with the dimensionality issue in a variety of different methods (for a review of these methods, see Nevo [2000]). Following Hausman, Leonard and Zona [1994] and Ellison, Cockburn, Criliches, *et* *al*. [1997], we use the notion of weak separability of preferences and multistage budgeting to estimate a series of flexible conditional demand functions. Using parameters of conditional demand systems, we then back out the unconditional elasticities.

For various stages of the multi-budgeting process, we estimate the Almost Ideal Demand System (AIDS) introduced by Deaton and Muellbauer [1980a,b1980b] which has several desirable properties. First, since the AIDS equations are based on a utility function of the generalized Gorman polar form (for a representative consumer), they satisfy the conditions for multistage budgeting (at least for the exact two-stage budgeting process). Second, the AIDS model aggregates well over consumers and provides an easy way of imposing theoretical restrictions, e.g., adding-up, homogeneity and symmetry (in the estimation procedure, we impose all three restrictions). Third, and most importantly, from an empirical standpoint, the AIDS specification provides a flexible substitution pattern between drugs within the same segment. The demand elasticities for individual drugs in a segment are not constant but functions of prices, and any pair of drugs in the system can be complements or substitutes. The resulting Engel curves are nonlinear, a desirable feature often noted in empirical studies. Finally, while the representative consumer metaphor is retained, the model can accommodate demographic effects, location, and time trends.

In the discrete choice literature, it is well known that imposing an arbitrary grouping and nesting structure for differentiated products can lead to unexpected results. Further, the results are not necessarily invariant to alternative grouping schemes. In the absence of a universal grouping rule, segmentation should be based on the unique features of the industry under study. We do so here on the basis of the pharmacological properties of these drugs discussed earlier.

### IV(i). Nesting and Specifications

Let there be *M* molecules indexed by *m* ∈{1,2,…,*M*}. For each molecule *m* there are *f* forms given by *f_m_* ∈{1,2,…,*F_m_*} and for each molecule *m* and form *f* there are *i* drugs given by 
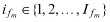
. For the ADHD drugs, there are four main molecules (*M* = 4). These are methylphenidates (MPH is *m* = 1), mixed amphetamine salts (MAS is *m* = 2), dextroamphetamines (DEX is *m* = 3) and all other molecules (OTH is *m* = 4). For the methylphenidates (*m* = 1), there are four forms: Immediate Release (IR), Extended Release Tabs (ER-TAB), Extended Release Caps (ER-CAP) and Osmotic Release Oral System (OROS). Note that in the ER-CAP segment, there are only two drugs, Metadate CD and Ritalin LA. The latter was introduced in 2002 and hence the segment can only be estimated using a maximum of two years of data. To overcome this data limitation, we pre-merged Ritalin LA with Ritalin SR to create a new drug ‘Ritalin SR/LA’ and Metadate CD with Metadate ER to create a new drug ‘Metadate ER/CD.’ The share of Ritalin SR/LA within MPH-ER is simply the ratio of the sum of revenue of SR and LA to the total segment revenue (and similarly for Metadate CD/ER) and hence the MPH segment now consists of only three forms and thus *f*_1_ = {1,2,3}.

**Figure 1:**
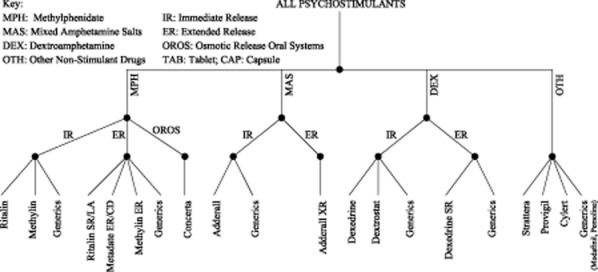
Taxonomy of ADHD drugs by Molecule, Form and Brand Names*Note:* Generics refer to several manufacturers for each molecule and form given in the column. There are no generic versions of Concerta and Adderall XR during the study period.

For the mixed amphetamine salts as well as for dextroamphetamines there are two forms each, Immediate Release and Extended Release (thus, *f*_2_ = {1,2} and *f*_3_ = {1,2}). The last group, (other molecules (*m* = 4)) consists of drugs with three separate molecules, modafinil, pemoline and atomoxetine. Only pemoline is available as both a branded (Cylert) and generic drug, while the other two are sold only as branded drugs in the U.S. (Modafinil and Strattera, respectively). We kept these three molecules in one category because they are very different from all other drugs considered so far. Strattera is a non-stimulant ADHD drug while pemoline and modafinil are stimulants, but because of their severe sides effects, none is considered a first line drug for ADHD and are often used for treating narcolepsy.^8^ Further, with the exception of pemoline, which is available as tablet and chewable tablet, these drugs are not available in alternative delivery mechanisms (the relative share of chewable pemoline tables in 2003 was only .01%). Hence, for this segment, there is only one form (i.e., *f*_4_ = {1}). The specific drugs within each molecule and form are summarized in Figure 1.

Using multistage budgeting we estimate demand parameters for each of the segments starting with the segments at the bottom level of the tree. The set of equations estimated are


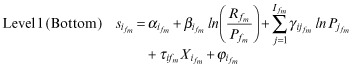
1a


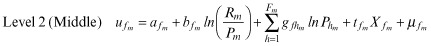
1b


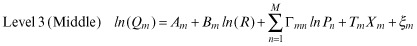
1c


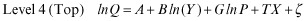
1d

where the equations at different levels are linked by the Stone price indexes (see Deaton and Muell-bauer [1980b]) given by


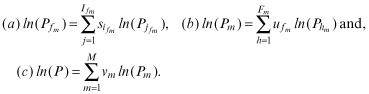
2

The bottom level segment consists of drugs in the same molecule and form. Thus, the share of the *ith* drug in molecule-form *f_m_* is given by 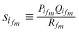 where 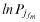 is the log price of the *jth* drug in form *f* within molecule *m* and there is a total of 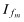 drugs in this molecule-form segment (and hence the number of share equations per segment is 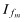 ). The terms 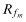 and 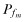 are the total revenue and ‘price’ of the *f* − *m* segment, where 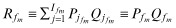 and the latter is constructed as the share-weighted sum of the (log) prices, i.e., the Stone-index given by equation (2). Similarly, 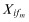 and 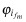 represent other exogenous variables and the error terms that affect shares in the segment. Note that there are two other implicit subscripts *a* and *t* that represent area and time. Thus, more accurately, 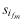 should be written as 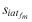 to mean the share of drug *i* in area *a* at time *t* within the molecule-form f-m. Similarly, the error term for observation from area *a* and period *t* is 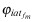. However for ease of exposition, we suppress these additional subscripts for now and discuss the stochastic specification of the equations and additional exogenous variables in a later section.

At the next level up (level 2), 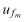 is share of the *fth* form in molecule *m*. The structure and meaning of variables in the level 2 equations is similar to the bottom level share equations. The variable 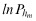 is the price of the form *h* in molecule *m* and is precisely the same term as the price index used in the bottom level equation. Further, *P_m_* is the ‘price’ of the molecule, constructed as the share-weighted Stone index of the price index of the forms within a molecule, and is given by the middle expression in equation (2). Level 3 consists of quantity equations in Cobb-Douglas form, i.e., *ln*(*Q_m_*) is the log-quantity of molecule *m* and is a function of log prices of the molecules *ln*(*P_n_*) which are the same variables as *P_m_* in equation (1b). Finally, at the top level (level 4) estimation involves a single equation in log form where total quantity *Q* is a function of total disposable income (*Y*) in the local area and *lnP* is the Stone index of the price over all *M* ADHD molecules constructed as the share-weighted average of the price indexes of the molecules (see last expression in equation (2) where *v_m_* is the share of each molecule).

### IV(ii). Identification

It is widely recognized that individual drug prices in a demand system are likely to be endogenous, requiring appropriate instrumental variable methods. One common approach to finding a set of valid instruments is to use the price of the product from another market based on the assumption that prices in different cities are correlated via common marginal cost shocks (see Hausman, Leonard and Zona [1994], Hausman [1997], Hausman and Leonard [2002], Nevo [2000, 2001]). The validity of these instruments hinges upon the assumption that there are no common demand side shocks across cities (see Bresnahan [1997]). For example, demand side errors could be correlated across cities due to regional or national level advertising campaigns, rendering the instruments invalid.

We use the same basic approach here for identification. We already have time trends and area dummies included in the specification. To reduce the possibility of common regional demand side shocks, we choose counties from far away regions to construct the instruments. Thus, for county *c* located in South census region, we draw 20 random counties from the other three remaining U.S. census regions and use the average price of the drug observed in the 20 counties as an instrument for the price of the drug in county *c* (we experimented with using 1,5,10 or 20 counties and the results were fairly similar). While using the average price from far away counties reduces the possibility of common demand side shocks due to regional effects, common demand side shock at national level (promotion by a manufacture in all media markets or a national ADHD awareness campaign) remains a possibility.

### IV(iii). Exogenous Variables

States differ in laws regarding the monitoring of psychostimulant drug consumption. For instance, some states require pharmacies to record and report to a local monitoring agency (such as the state DOJ) each script filled out for a psychostimulant drug along with identifying information about the prescribing physician and some demographic information about the patient. Such laws can potentially affect retail level price (due to monitoring cost), and the presence of reporting laws in a state may correlate with the demand/share of individual drugs since the physician may be concerned about being flagged as prescribing controlled substances ‘too much.’ States also differ substantially in school accountability laws and there is some evidence that accountability laws are correlated with the diagnosis of ADHD and the consumption of psychostimulants (Bokhari and Schneider [2011]). Similarly a major driver of ADHD drugs is access to Medicaid, which varies by state and year. Many states (at varying rates) also carved-out their mental health benefits (including ADHD) to specialty carve-out firms and Ling, Berndt and Frank [2008] show that this affected demand for various psychotropic drugs. For these and other similar concerns we include state level variables (state Medicaid population and Medicaid drug expenditures which vary by year) as well as state level dummies in all regressions. As additional controls we also include the log of number of physicians and the log of children in a county in all specifications. Since either ‘taste’ (or general acceptance) for a specific drug or a type of drug may be changing over time, we also include up to a cubic polynomial in time in each segment. If the segment was estimated for less than four years, or if the cubic term was not significant, we would only include up to a quadratic term so as to avoid problems of multi-collinearity. County level variables, such as employment rates, per capita income, and other demographic variables by race, etc., were also added in alternative specifications and are reported in the robustness section.

Finally, we also include in all level 3 Cobb-Douglas equations the proportion of 12-hour drugs in the MPH and MAS segments. These variables are included because the Cobb-Douglas equations are quantity equations for four aggregate molecules whose characteristics are changing over time at different rates. If consumers derive utility from a product providing 12-hour coverage, then these characteristics would also affect demand and relative choice among molecules independent of the price effects. For instance, the combined share of all MPH drugs declined from 50% in 2000 to 37.6% in 2003 while the share of all MAS drugs increased from 31% to 34%. Over the same period the average price of MPH molecule increased from roughly $47.9 to $76.3 while the average price of MAS increased from $19.8 to $43 (adjusted for dosage differences). Put another way, in 2000, the share of MPH was 1.61 times that of MAS when it was 2.4 times more expensive, but in 2003, its share was 1.08 times that of MAS even though now it was only 1.78 times more expensive. One possible explanation for the change in shares of molecules relative to the change in average prices is the proportion of the 12-hour drugs within each molecule: in 2000, the share of the 12-hour drugs within MPH was 9.3% and the share of the 12-hour drugs within MAS was 0% but by 2003, the share of 12-hour drugs within the two molecules was 69.4% and 69.3% respectively (Concerta was introduced in 2000 and Adderall XR was introduced in 2001, the only 12-hour drugs in the two molecules respectively). Thus, we control for the changing characteristics of these molecules by including the proportion of 12-hour drugs within each of these molecules.

### IV(iv). Other Estimation Issues

When a patient switches from one drug to another, the conversion is not always gram for gram. Thus we converted quantities and prices from grams to defined monthly dosage using the medication equivalence given in Table 1. The conversion algebra, along with other estimation issues are described in Appendix A and include: (a) the use of year specific area-averaged shares in the construction of price indexes in equation (2), (b) the system estimation for each segment separately, (c) homogeneity and symmetry restrictions, and (d) the use of bootstrap methods to obtain standard errors.

## V. Results

### V(i). Quantity Equations—Top and Middle (Levels 4 and 3)

We begin with the results of the top and molecule level equations, reported in Table III. The table shows selected coefficients from the OLS and IV estimation (the remaining coefficients are given in Appendix B). The OLS estimate of price elasticity in the top equation is −2.2 and the IV estimate is −1.2 and both are statistically significant at the 5% level. If simultaneity were the only source of endogeneity in the equation, one would expect the OLS estimate to be smaller in magnitude than the IV estimate. However, the top level equation is for all ADHD drugs combined, and several other variables that may affect demand (e.g., social capital, churches per capita, school accountability laws, etc.) are excluded from the equation and are in the error term. The direction of OLS bias depends on whether *cov*(*u_i_*, *w_i_*) is positive or negative (where *u_i_* and *w_i_* represent the error term and the exclude variables from the equation). In particular, if *cov*(*u_i_*, *w_i_*) < 0 then under OLS, 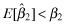 where *β*_2_ represents the coefficient on price. This could happen for instance, if the strength of school accountability laws is positively correlated with demand for ADHD drugs but negatively correlated with the price.

**Table III:** Selected Coefficients—Top & Middle Level (Log) Quantity Equations

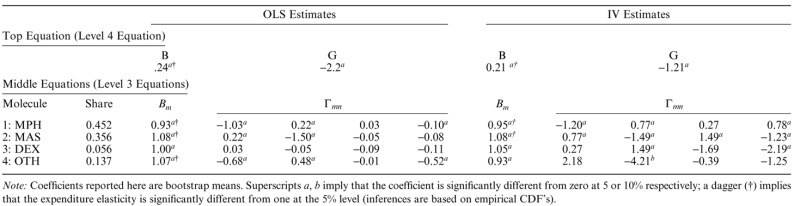

Estimates of level 3 quantity equations are also shown in the same table. The expenditure elasticities for all four molecules range from .93 to 1.08 under OLS and IV. For the MPH and MAS molecules, they are also statistically different from one, i.e., demand is not homothetic for these two molecules. However, a further joint test of all four expenditure elasticities being one is rejected (joint homotheticity tests for each segment in the IV estimations are given in Appendix B). There are considerable differences in the price elasticities of OLS versus IV, where the IV estimates of the conditional demand functions are typically larger in magnitude than the OLS estimates. We restrict further discussion to the IV estimates. The off-diagonals of 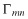 provide the Marshallian cross elasticities (the number in row *m* column *n* is the elasticity of drug *m* with respect to a price change in drug *n*). We expect off-diagonals to be positive, or at least not significant when negative, since the molecules would be either gross substitutes or possibly not related in cross-price effects if the molecules cannot be exchanged therapeutically. This turns out to be true among the three main molecules MPH, MAS and DEX—drugs that generally cannot be mixed—but some complementarity is indicated with drugs in the ‘OTH’ category. This result could be either due to inappropriate aggregation of three very different molecules (pemoline, modafinil and atomoxetine) into a catch all group ‘OTH'—a modeling choice made necessary due to data limitations—or indicative of the simultaneous use of main ADHD drugs and other drugs. The pharmacological literature on ADHD suggests that 4–5% of patients (and 8–10% among some age groups) have their drug treatment ‘augmented’ with an additional ADHD drug where the augmentation could be either with a drug in the same or another class of drugs and would explain the complementarity with the ‘OTH’ group (Perwien, Hall, Swensen, *et* *al*. [2004], Christensen, Sasan, Hodgkins, *et* *al*. [2010], Hodgkins, Sasan, Christensen, *et* *al*. [2011]). Nonetheless, the fourth equation is not estimated very precisely and we discuss it further in the robustness and sensitivity section.

### V(ii). Share Equations—Forms within Molecules (Level 2)

Next we discuss the results from the middle level share equations for forms within each of the first three molecules (not including the fourth molecule since drugs in OTH are conglomerated into one drug). Since the middle and bottom level share equations are in AIDS form and involve prices as well as the price index across forms, interpreting the estimated coefficients is more complex. Thus, rather than discuss the regression coefficients, we provide and discuss the estimated elasticities, computed at average shares, in Table IV. Also, we restrict our discussion to results based only on 3SLS estimates (henceforth referred to as IV estimates). The conditional elasticities (conditional on *R_m_*) of forms within a molecule with respect to the ‘price’ of the form is derived in Appendix A and is given by


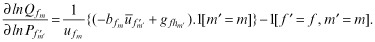
3

Within the MPH molecule, the own price elasticities of all three forms are elastic and that of Concerta is −2.92. Concerta appears to be a strong substitute for the other two forms (all estimates are statistically significant). However, the immediate release and extended release forms are both gross and net (Hicks-Allen) complements of each other. This result suggests that children are often simultaneously using ER and IR: the extended release version is taken in the morning before going to school (lasting about 8 hours) and a short-acting immediate release version is taken after school (which lasts about 4 hours) to carry them over to the evening. However, such a mixture is not needed with the OROS/Concerta (which lasts 12 hours), and hence it acts as a substitute for the other two forms. Next, within the MAS molecule, both the ER (i.e., Adderall XR which lasts about 12 hours) and IR versions (4-hour) are price elastic and net/Hicksian substitutes. Unlike MPH-ER and MPH-IR, Adderall XR is not taken in combination with the immediate release version and hence MAS-ER and MAS-IR are net substitutes rather than complements. Nonetheless, the gross substitution pattern is not as large in magnitude nor statistically significant. This is perhaps because the 12-hour medication cannot be easily substituted with three dosages of a 4-hour medication since one of the dosages would need to be taken midday and may be difficult in some school settings.

**Table IV:** Conditional Elasticities—Middle Level (Form within Molecule)

	Marshallian Elasticity			Hicks-Allen Elasticity			Expenditure Elasticity	Average Share
MPH: Molecule 1. Share among ADHD drugs is .452								
	(A)	(B)	(C)	(A)	(B)	(C)		
(A) MPH-IR	−1.97*^a^*	−0.49*^a^*	1.54*^a^*	−4.97*^a^*	−1.81*^a^*	4.11*^a^*	0.93*^a†^*	0.33
(B) MPH-ER	−0.94*^a^*	−2.34*^a^*	2.28*^a^*	−1.81*^a^*	−11.94*^a^*	5.71*^a^*	1.00*^a^*	0.18
(C) MPH-OROS	1.02*^a^*	0.84*^a^*	−2.92*^a^*	4.11*^a^*	5.71*^a^*	−4.97*^a^*	1.05*^a†^*	0.48
MAS: Molecule 2. Share among ADHD drugs is .356								
		(D)	(E)		(D)	(E)		
(D) MAS-IR		−1.25*^a^*	0.30		−1.15*^a^*	1.68*^a^*	0.95*^a†^*	0.59
(E) MAS-ER		0.36	−1.43*^a^*		1.68*^a^*	−2.45*^a^*	1.07*^a†^*	0.41
DEX: Molecule 3. Share among ADHD drugs is .056								
		(F)	(G)		(F)	(G)		
(F) DEX-IR		−0.93*^a^*	0.02		−2.03*^a^*	0.94*^a^*	0.91*^a†^*	0.32
(G) DEX-ER		−0.03	−1.01*^a^*		0.94*^a^*	−0.44*^a^*	1.04*^a†^*	0.68

*Note:* Coefficients reported here are bootstrap means. Superscripts *a*,*b* imply that the coefficient is significantly different from zero at 5 or 10% respectively and a dagger (†) implies that the expenditure elasticity is significantly different from one at the 5% level (inferences are based on empirical CDF's). Molecule 4 is the all other ADHD drugs group and we do not distinguish between forms. Hence, no middle level share equations exist for molecule 4. MPH-OROS is Concerta and MAS-ER is Adderall XR.

In the DEX class each of the forms exhibits unit elasticity. No firm conclusion can be drawn about the substitution patterns across forms. The DEX group consists of three drugs in the IR form (4-hour) and two drugs in the ER form (8-hour). These forms could be substitutes (a 4-hour medication taken twice a day instead of a single 8-hour dose), complements (one 8-hour medication followed by one 4-hour medication), or not related. Our data cannot differentiate between these patterns.

### V(iii). Share Equations—Drugs within Molecule-Forms (Level 1)

Finally, we provide the elasticities of individual drugs within their respective molecule-forms in Table V. Starting with the MPH-IR segment, the own price elasticities of Ritalin and generics are either in the elastic region or not statistically different from one, but that of Methylin is inelastic. Further, Methylin and generics are substitutes for Ritalin, both gross and net, but they appear to be gross complements of each other. *A priori*, we expect all three drugs to be substitutes—since they consist of the same molecules and are in the same forms. However, the complementarity is not statistically significant when the income effect is held constant as indicated by the Hicksian elasticities.

**Table V:** Conditional Elasticities—Bottom Level (Drugs within Molecule-Form)

	Marshallian Elasticity				Hicks-Allen Elasticity				Exp Elasticity	Avg Share
MPH-IR: Molecule 1, Form 1. Share within Molecule is .33										
	(1)	(2)	(3)		(1)	(2)	(3)			
(1) Ritalin	−2.13*^a^*	0.28	0.79*^a^*		−9.44*^a^*	2.24*^b^*	2.47*^a^*		1.06*^a^*^†^	0.20
(2) Methylin	0.25	−0.39	−0.89*^a^*		2.24*^b^*	−0.61	−0.55		1.03*^a^*	0.24
(3) Generics	0.30*^a^*	−0.36*^a^*	−0.91*^a^*		2.47*^a^*	−0.55	−0.66*^a^*		0.97*^a^*^†^	0.56
MPH-ER: Molecule 1, Form 2. Share within Molecule is .18										
	(4)	(5)	(6)	(7)	(4)	(5)	(6)	(7)		
(4) Ritalin SR/LA	−3.11*^a^*	0.32	1.05*^a^*	0.73	−11.21*^a^*	2.22*^a^*	11.71*^a^*	2.91	1.01*^a^*	0.25
(5) Metadate ER/CD	0.26	−1.94*^a^*	−0.00	0.49*^b^*	2.22*^a^*	−6.21*^a^*	1.19	2.47*^a^*	1.19*^a^*^†^	0.26
(6) Methylin ER	2.75*^a^*	0.07	−3.18*^a^*	−0.56	11.71*^a^*	1.19	−31.47*^a^*	−0.53	0.92*^a^*^†^	0.10
(7) Generics	0.52	0.42*^a^*	−0.14	−1.68*^a^*	2.91	2.47*^a^*	−0.53	−3.47*^a^*	0.88*^a^*^†^	0.39
MAS-IR: Molecule 2, Form 1. Share within Molecule is 0.59										
	(9)	(10)			(9)	(10)				
(9) Adderall	−2.30*^a^*	1.34*^b^*			−4.4*^a^*	3.32*^a^*			0.96*^a^*^†^	0.43
(10) Generics	0.98	−2.01*^a^*			3.32*^a^*	−2.50*^a^*			1.03*^a^*^†^	0.57
DEX-IR: Molecule 3, Form 1. Share within Molecule is .32										
	(12)	(13)	(14)		(12)	(13)	(14)			
(12) Dexedrine	−4.28*^a^*	2.31*^a^*	1.03		−14.58*^a^*	6.71*^a^*	4.11		0.94*^a^*^†^	0.28
(13) Dextrostat	1.57*^a^*	−2.78*^a^*	0.18		6.71*^a^*	−5.91*^a^*	1.59		1.03*^a^*^†^	0.40
(14) Generics	0.85	0.23	−2.10*^a^*		4.11	1.59	−5.45*^b^*		1.02*^a^*	0.32
DEX-ER: Molecule 3, Form 2. Share within Molecule is .68										
	(15)	(16)			(15)	(16)				
(15) Dexedrine SR	−1.66	0.67			−1.92	2.55			0.99*^a^*	0.57
(16) Generics	0.88	−1.89			2.55	−3.39			1.01*^a^*	0.43

*Note:* Coefficients reported here are bootstrap means. Superscripts *a*, *b* imply that the coefficient is significantly different from zero at 5 or 10% respectively and a dagger (†) implies that the expenditure elasticity is significantly different from one at the 5% level (inferences are based on empirical CDF's).

In the next segment (MPH-ER), there are four drugs and each has an elastic demand. While the three branded drugs are gross substitutes for each other, once again Methylin ER and the generics appear to be complements (but not statistically significant).

As noted earlier, the price of Methylin ER was typically mid-way between the price of the generics and Ritalin SR and initially the price of Metadate CD was lower than that of Ritalin LA. Further, the price of Methylin ER was always much lower than Ritalin SR and only a few dollars more than that of the generics. Since Metadate ER/CD and Methylin ER are ‘brands’ they offer some (perceived) quality enhancement over the generics, but since they are priced between the price of Ritalin SR/LA and the generics, they offer some advantage compared to Ritalin SR/LA. The substitution patterns reveal that by entering as low-priced brands, they were able to siphon off demand from Ritalin SR/LA as well as from the generics: a price increase in Ritalin SR/LA leads to consumers' switching mostly to Methylin ER rather than to generics and similarly, a price increase in generics leads to consumers switching to Metadate ER/CD (see columns (4) and (7)).

In the next three bottom level segments (MAS-IR, DEX-IR and DEX-ER), own demand elasticities for all medications are elastic but not significant for DEX-ER. The substitution patterns suggest that these drugs are gross and net substitutes for other drugs within their own molecule-forms or possibly not related when not significant. However, it should be noted that the segments MAS-IR and DEX-ER are both estimated with only two years of data (see Table II) and the relative lack of significance on cross elasticities between, say Adderall and generic Adderall, may reflect a lack of statistical power rather than that these drugs are not gross substitutes for each other. In fact, with the exception of Methylin and generic MPH-IR, all drugs are either substitutes or not related to other drugs in their own molecule and form as expected but could be complements with drugs outside the segment.

### V(iv). Restrictions Tests

Within each level 1 and level 2 segment, we imposed and tested the homogeneity and symmetry restrictions (jointly via the Wald statistic). The null of valid restrictions was rejected in two segments (MPH-IR and MAS) at the 1% level and four segments at the 5% level (the results of the test are given in Appendix B). Because these restrictions are implied by theory and without them it does not make sense to proceed with any welfare calculations (since then estimated parameters do not necessarily correspond to any utility functions) we continued to impose homogeneity and symmetry in all level 1 and level 2 equations.

### V(v). Unconditional Elasticities

While the multi-budgeting process allows estimation of the conditional demand functions, the cross-price effects are limited to within the molecule-form segment. Unconditional effects are more general and include the induced demand effects that work through the budget (expenditure) shares among all drugs, inside and outside the *f* − *m* segment. Thus, a drug that introduces an important new variety may have a widespread consumption impact across all segments; its introduction may induce a substantial demand response in patients (and their providers) who earlier had been using scripts chosen from any one of the ADHD drugs. In the absence of the full unconditional demand system, it is still possible to assess the broader effects of one drug onto another (at least locally) by estimating the unconditional elasticities from parameters of the conditional demand systems. The unconditional elasticity (derivation given in the appendix) is computed from the parameters of the conditional demand functions as


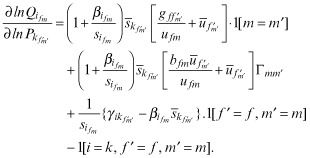
4

The unconditional elasticity estimates from our IV estimates for selected drugs, those in the MPH and MAS segments are shown in Table VI (the full 17 by 17 unconditional elasticity matrices under IV and SUR estimations are in the appendix). The estimates in this matrix are generally consistent with earlier results, particularly where they were significant. For instance, unconditionally, drugs 1 and 3 are gross substitutes to each other whereas Methylin and generic MPH-IR are gross complements, drugs 4−7 have the same sign patterns as before (at least where significant), drugs 1−3 are complements to drugs 4−7 and drugs 1−7 are substitutes for drug 8 (consistent with MPH-IR and MPH-ER being complements and both being substitutes for OROS). Similarly, in terms of relative magnitudes of cross-elasticities, while drugs 9 and 10 are substitutes for each other (4-hour MAS drugs), neither is a strong substitute for drug 11 (12-hour MAS drug). The substitution patterns outside the molecule are also consistent with the overall substitution patterns across molecules observed earlier in Table III.

**Table VI:** Unconditional Elasticities from IV estimates

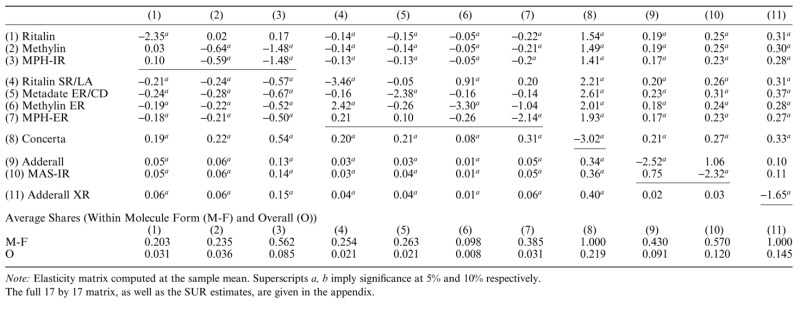

Since the Marshallian elasticities are not symmetric, it is useful to discuss the unconditional matrix explicitly in terms of a price change in drug *i* on the demand for drug *j* and vice versa. Consider first the effect of price changes of the new entrants on the demand for other drugs. A price increase in Metadate ER/CD and Methylin ER (columns 5 and 6 respectively) results in either an increased demand for other drugs in the same molecule-form segment, as also suggested by the conditional elasticities in MPH-IR segment, or possibly no effect since unconditional elasticity with generic MPH-ER is not significant. Outside the segment, it leads to an increased demand for Concerta and MAS drugs but not for any of the 4-hour MPH-IR medications where consumption would decrease due to complementarity with them. On the other hand, a price increase in Concerta results in an increased demand for all the 4-hour and 8-hour medications in the MPH group as well as for drugs in the MAS group. Also note that the increase in demand for any of the MAS drugs, compared to an increase in the demand for MPH drugs, is relatively small for a price increase in any of the three MPH drugs. These patterns suggest that children on an MPH-ER/MPH-IR combination therapy mostly stay with the same mixed therapy after a price change due to the availability of choice, but those on a single dose of the 12-hour Concerta may switch over to a mixture of MPH-ER/MPH-IR, or even MAS, after a price increase in Concerta. By comparison, a price increase in either generic Adderall or Adderall XR (see columns 10 and 11) significantly increases the demand for drugs in the MPH segment suggesting a switch in choice of molecule, especially for a price increase in Adderall XR (as noted earlier, the magnitude of cross-elasticity between generic Adderall and Adderall is large and likely not significant only because these two drugs were estimated with two years of data). The switch from Adderall XR to MPH therapy could happen because a price increase in 12-hour Adderall XR leaves little choice within the molecule if 12-hour coverage is important, and hence patients either switch to the 12-hour MPH (Concerta) or an 8-hour/4-hour combination available in MPH. Next, reading row-wise, the results suggest that the demand for Metadate ER/CD, Methylin ER, and Concerta (rows 5,6 and 8) is affected by price changes in MPH and MAS drugs and the magnitudes are large. The result is particularly noteworthy for Concerta where a price increase in any of the other MPH or MAS drugs is associated with an increase in treatment via Concerta. By comparison, demand for generic Adderall and Adderall XR (rows 9 and 11) also increases with a price increase in any of the MPH based drugs, but the magnitude is much smaller compared to the ones in rows 5, 6, and 8.

These results suggest the following: removing Metadate ER/CD or Methylin ER from the choice set would result in children being switched to other drugs within the class or Concerta, implying some welfare reduction due to a switch to higher priced Concerta or to lower (perceived) quality of generic MPH-ER. On the other hand, removing Concerta from the choice set would result in possibly a larger welfare reduction since children are switched from a once a day Concerta to a mixed therapy option of MPH-ER and MPH-IR or switch to MAS therapy. Similarly, removing Adderall XR or generic Adderall switches children to non-MAS therapies which can lead to large welfare reductions. These effects are further complicated when one considers that the removal of any one drug from the choice set may also mean a change in the prices of the remaining drugs. For instance, if firms set prices as Nash-Bertrand, then removal of Concerta from the choice set (by exogenously setting its price high enough so that demand is zero) would imply a higher price of all remaining drugs due to the positive cross-elasticities in column 8. On the other hand, a higher price of Metadate ER/CD does not necessarily imply a higher equilibrium price of all remaining drugs since Metadate ER/CD is a complement to several drugs (see column 5). The next section computes the relative magnitudes of these effects.

### V(vi). Welfare Calculations

In this section, we report the estimated welfare changes associated with each of the new drug introductions: Concerta, Adderall XR, MAS-IR (i.e., generic Adderall), Methylin ER and Metadate ER/CD. Following several previous studies, e.g., Hausman [1997], Hausman and Leonard [2002, 2005], we compute the compensating variation associated with each new product by calculating the ‘virtual price,’ i.e., an artificial price for the new drug that would be just high enough to set the quantity demanded to zero. The virtual price is then used to simulate consumer welfare associated with the change in all prices from the pre- to post- introduction period. The virtual price for each new drug is inferred as an out-of-sample projection from the empirical demand parameter estimates. In particular, we use the unconditional elasticity matrix to back out the parameters of the unconditional demand system and use these parameters to solve for the price that would set the demand for the drug in question to zero. Let this be the virtual price of the drug prior to its introduction. The virtual price minus the observed price represents the hypothetical price difference in a ‘but-for’ world where the drug is first absent from the set of ADHD drug choices and then introduced in the market.

The computation algorithm for the new prices in the ‘but-for’ world is fairly standard and we only briefly describe it here. Using the unconditional elasticities and a Nash-Bertand price competition model, we first back out the marginal costs (*c_i_*) of each of the *I* products using the equation


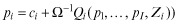
5

where 
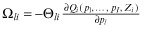
 and 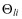 is a 1/0 matrix with ones in the leading diagonal and in locations when a firm jointly produces drugs *l* and *i* (for a complete derivation see Nevo [1998]). Next, using the virtual price of the drug of interest, the marginal costs estimated above, and the demand parameters, we solve for the equilibrium price of the remaining *I* − 1 products in each market (two computational adjustments were necessary and are described in Appendix A) This set of new prices can then be used to compute the *CV* using all the old observed prices and the new vector of prices.

Our welfare measure of compensating variations *CV* can be computed from the expenditure functions derived from the estimated top-level equation. Let the price vector change from **p***^o^* to **p**′ such that the price index (at the top level equation) changes from *p^o^* to *p*′. Then


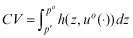
6

where


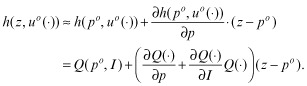
7

Prior to the calculations, there are two related issues in terms of interpreting the welfare calculations that need to be addressed. First, do patients have any sovereignty over the choice of a drug or do they only follow the brand/generic choices prescribed by the physician, who may not be price sensitive, and even if patients do, are they (or physicians as their agents) sensitive to price differentials given that most are insured and only make a co-payment? Insurance companies make extensive use of multi-tiered pharmacy benefits in their formularies where drugs placed in tier one often have a small co-payment by the consumer, those in tier two require substantially greater co-payment while tier three drugs require the highest co-payment. Recent evidence from the implementation of multi-tiering in different health plans suggest that even insured patients are sensitive to price differentials. Total expenditures, and more importantly, the utilization of medication, is found to be significantly lower for tier three drugs than for tier one preferred drugs (Huskamp, Deverka, Epstein *et* *al*. [2005, 2003,] Fairman, Motheral and Henderson [2003], Nair, Wolfe, Valuck *et* *al*. [2003], Rector, Finch, Danzon, *et* *al*. [2003], Thomas, Wallack, Lee *et* *al*. [2002], Motheral and Frirman [2001]). Thus, price differentials and/or discounts from manufacturers to insurance companies dictate the insurance companies' decision to place a drug within a specific tier which in turn appears to change the patient (or their doctor's) behavior to switch to cheaper drugs.

Second, given that both the patient and the insurance company make a payment, how should one interpret the area under the demand curve? The representative consumer metaphor used in this paper makes the decision process a joint decision by both the patient and the insurance company. The area under a representative consumer's demand curve is the sum of the consumer's surplus plus the insurance company's surplus and without explicit data on co-payments by each sale, it cannot be separated into the two components. Nonetheless, the sum of the two provides a useful way of accessing and comparing the net value of new introductions to society. Further, while savings from the consumption of a cheaper drug are initially made by the insurance company, in the long run and with a fairly large number of insurers, it can be argued that these would be passed onto the consumer in the form of lower premiums as well as lower co-payments if the patient chooses a drug in tier one. Thus, the *CV* computations given below are best used to judge the value of new introductions—generics and me-toos—to the society as a whole (insurer plus consumer) rather than how much of it is captured by different parties (for a similar interpretation of welfare calculations see Branstetter, Chatterjee and Higgins [2011, p. 21]).

Using 2003 data, we calculate the *CV* separately for each MSA county in the sample. The resulting distribution of estimates is reported in Table VII under the heading ‘Full Elasticity Matrix.’ The results are expressed in terms of total dollars in the locality, as a percentage of total ADHD expenditures in the county and in terms of ‘per-ADHD-child.’ The per-ADHD-child estimate is a crude approximation based upon the local non-adult population and a conservative estimate (5%) of the incidence of ADHD among children and adolescents. The welfare effects of each drug span a wide range across cities, reflecting unique local conditions in the consumption choices.

**Table VII:** Compensating Variation

Total CV Per County ($1000s)						
	Full Elasticity Matrix			Reduced Elasticity Matrix		
	Mean	Median	Std Dev	Mean	Median	Std Dev
Adderall XR	−369.36	−178.50	583.80	−356.41	−172.80	564.62
Concerta	−401.89	−200.84	584.32	−384.27	−193.32	556.55
MAS-IR*	−201.14	−93.46	324.81	−133.64	−61.31	218.50
Metadate CD/ER	−52.15	−25.56	79.32	−47.39	−22.95	71.72
Methylin ER	−14.96	−6.63	25.99	−15.82	−6.86	26.27
CV as Percentage of Total Expenditure Per County						
Adderall XR	−14.41%	−14.26%	3.66%	−13.89%	−13.80%	3.67%
Concerta	−16.65%	−16.53%	3.58%	−15.99%	−15.80%	3.57%
MAS-IR*	−7.74%	−7.60%	2.44%	−5.08%	−4.91%	2.05%
Metadate CD/ER	−2.15%	−1.99%	0.86%	−1.96%	−1.81%	0.81%
Methylin ER	−0.62%	−0.53%	0.41%	−0.67%	−0.58%	0.42%
CV per ADHD Child in County ($/per ADHD Child)						
Adderall XR	−123.38	−104.11	93.10	−119.23	−100.06	90.88
Concerta	−137.04	−120.94	92.39	−131.41	−115.83	88.79
MAS-IR*	−65.18	−54.82	49.27	−43.47	−34.97	36.01
Metadate CD/ER	−17.32	−15.02	11.94	−15.78	−13.61	10.97
Methylin ER	−4.67	−3.93	3.50	−5.04	−4.27	3.74

* MAS-IR is generic Adderall.

*Note:* The table displays the CV in a ‘but for’ world where the listed drug is not in the choice set (i.e., it is priced at the virtual price) and all remaining *N* − 1 drugs are at a new NB price equilibrium. The second set (under ‘Reduced Elasticity Matrix’) is the CV calculations when all non-significant cross-elasticities are set to zero.

The results reveal that the largest welfare benefits were generated by three drugs, Concerta, Adderall XR, and generic Adderall. The introduction of generic Adderall led to significant market expansion while the other two were the first 12-hour drugs within their respective molecules. In total, these three drugs accounted for 57.6% of the ADHD market in 2003. Outside of drugs in the OTH group (mostly Strattera), none of the other drugs in our sample have as much as a 5% share. The introduction of Concerta produced the largest estimated welfare effect, followed by that of Adderall XR and generic Adderall. On average, the welfare gain associated with Concerta is $401K, or about $137 per ADHD child. The estimated value has large variations across the country. This variation is partly due to the population size, with the largest cities generating up to $5.89 million in compensating variations overall. Expressed as dollars per ADHD child, the range of values ($924 to $3) is over two hundred fold. This suggests that there are considerable local area variations in the acceptance of these drugs. In contrast, the effects of the other two drugs, Metadate ER/CD and Methylin ER—introduced in segments where several branded and generics already existed—are much smaller, i.e., consumers derive relatively less benefit from the increased choices they provide.

Several cross elasticity estimates, particularly in the DEX segment, were not significant. To check the sensitivity of welfare calculations to non-significant cross price elasticities, we re-estimated the N-B equilibrium prices and the CV computations by first setting non-significant off-diagonal elasticity terms to zero. The results are given in the column ‘Reduced Elasticity Matrix’ and show that while the point estimates are different, the magnitude, variation and the relative ranking do not change.

### V(vii). Robustness and Sensitivity

The reported estimates in the previous sections were generally robust to several small changes in specifications or estimation procedures. Adding or dropping exogenous variables such as (log of) number of MD's, number of children, Medicaid population or public drug expenditures by states did not change the estimates in any substantial way. We also estimated models with additional county level variables (demographic breakdowns by race, per capita income, unemployment rates, etc.) with no significant changes in the estimated elasticities. Changes in estimation procedures such as switching from full system estimations (3SLS or SUR) to partial system estimations (i.e., 2SLS and OLS but with cross-equation and within-equation restrictions imposed by the homogeneity and symmetry conditions) did not affect the estimated parameters much. The results were also robust to how the Stone price index was constructed within each segment. Within each segment, we used year-specific area-averaged shares 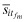 in the construction of the price index but switching to area-specific year-averaged shares 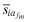 did not change the results.

One exception to the robustness was the ‘OTH’ drug estimates in the level 3 equations shown in Table III. The share of ‘OTH’ increased dramatically in 2003 when Strattera—the only non-stimulant ADHD drug—was introduced. The share of ‘OTH’ (the aggregate drug 17) increased from 10% in 2002, when it consisted of only pemoline and modafinil molecules to 25.6% in 2003 when it also includes the branded version of atomoxetine (Strattera) without much change in the price of ‘other drug.’ Further, this large increase was almost entirely due to Strattera (Table II) which, on average consisted of 61% of the ‘OTH’ drug and ranged from 19% to 99% across counties. We experimented with accounting for the introduction of a non-stimulant drug (which changes the nature of the ‘OTH’ drugs as all others are stimulants), by removing Strattera from the ‘OTH’ group, dropping 2003 data, adding a dummy variable for Strattera, adding the relative share of Strattera within ‘OTH,’ and by entirely removing the ‘OTH’ group from level 3 equations. Each of these changes only reconfirmed that the ‘OTH’ equation, and consequently the own and cross elasticities of drug 17 in the unconditional elasticity matrix, are not robust (the others were fairly stable). Thus in our final estimate, we did not impose any homogeneity in the level 3 Cobb-Douglas equations, nor did we impose any symmetry restrictions between ‘OTH’ and the other three molecules and used single equation methods to estimate this segment. Finally, since the cross-effects of this last drug are large and in turn affect the welfare computations, in the welfare calculations we set the off-diagonal in the elasticity matrix associated with this drug to zero. This was so that when the Nash-Bertrand equilibrium prices are computed for the remaining 16 drugs in the but-for world, the cross-effects with this drug do not impact those calculations. Thus, in the but-for world calculations given earlier, the price of this other drug does not change nor does it interact with the remaining 16 drugs.

### V(viii). Alternative Nesting

Our current nesting is based on the grouping of drugs given in Table I and that the decision maker first chooses a molecule and then the form. Alternatively, if the decision maker first chooses the form (4-hour, 8-hour, 12-hour, or other) and then the molecule, followed by the choice of specific drugs, it would still result in the same bottom level (level 1) grouping as well as the same top level equation (tree diagram omitted). However, the level 2 share equations would consists of shares of molecules within forms (e.g., in the 12-hour form, Adderall XR and Concerta would be the two drugs in this segment) while level 3 would consist of Cobb-Douglas equations for quantities by form rather than quantities by molecules. We estimated all equations under this alternative tree structure as well. The middle two level equations (level 2 and level 3) under the alternative tree structure are not directly comparable to the original estimates but several results from this alternative structure seemed implausible, both because of the implied substitution patterns and the resulting upward sloping demand curves for several drugs. We interpret these alternative results as indicating that the initially imposed structure—molecule followed by the form rather than the other way round—is consistent with the observed data. While it is possible that some physicians/consumers may first be choosing the form and then the molecule, perhaps the majority and hence the typical decision maker chooses the molecule followed by the form.

### V(ix). Limitations

We turn now to note potential limitations of this study and its results. First, our data on drug sales at the pharmacy level omit unreported payments in the form of rebates directly to the payer, e.g., the Medicaid Rebate Program. The net effect of these adjustments on payment flows to pharmacies, i.e., the proportion omitted from our data, is difficult to trace due to diverse methodologies used by states for reimbursing pharmacies. Second, with the data available for this study, we were not able to measure the effect of one important new drug, Strattera. It entered in the final year of our panel and we had to aggregate it into a collective set of other drugs.

Third, pharmaceutical products in general, and certainly ADHD drugs in particular, are experience goods and we should expect high marketing-to-sales ratios for these drugs (Nelson [1974]). Indeed total promotion to manufacturer sales were at 14% in 2000 (Frank, Berndt, Donohue *et* *al*. [2002]). If the marketing-to-sales ratios of drugs analyzed here are fairly similar (at least for some of the block buster drugs) then given that we estimate large elasticities for Concerta, Adderall XR, etc., the Dorfman-Steiner theorem would imply that the advertising elasticities are also fairly large. Nonetheless, we do not have promotional activity data by specific drugs and markets and hence cannot estimate advertising elasticities nor can we provide any insight into how promotional activities affect substitution possibilities within or across the molecules and forms. While it would be interesting to know how changes in direct-to-consumer marketing and physician detailing affected market shares in the ADHD market, that question is beyond the scope of this study.

## VI. Summary and Discussion

The models and methods employed here with the aid of richly detailed data are effective tools for evaluating drug demand systems in pharmaceutical markets like ADHD drugs, where large sets of differentiated products experience episodes of new drug introduction. Our demand analysis shows that the demand for ADHD drugs is elastic and there are significant substitution possibilities among these drugs, both within the molecule and form as well as across segments. Further, it sheds light on why some drugs were more successful than others. Both Concerta and Adderall XR created new niche markets within their respective molecules by introducing new delivery mechanisms. Consumers placed a large value on these introductions, on average approximately $137 and $123 per child per year respectively, and consequently these two drugs achieved 24% and 26% of the ADHD drug market. The introduction of generic Adderall in the MAS-IR segment extended the market and was also very valuable to consumers (about $65 per child per year). Further, these three drugs are substitutes for other drugs and consequently their introduction led to lower equilibrium prices of other drugs. However, the two other introductions, Methylin ER and Metadate CD, did not create new niche markets (albeit Metadate CD was the first to provide a combination of rapid-release and slow-release beads via a capsule in the MPH-ER segment) since both were introduced in a segment where branded as well as generic drugs already existed. Further, being complements to drugs in the MPH-IR segment, their introduction is associated with a higher equilibrium price for drugs in the MPH-IR segment. Consequently, consumers placed a lower value on these introductions, as measured by the welfare calculations, which may explain the low market shares of these two drugs (.6% and 2.6% respectively).

Our results speak directly to the policy proposals aimed at slowing the introduction of me-too drugs (Angell [2004], Goozner [2004]). Angell [2004] calls upon the FDA to change its approval standards and require me-too drugs to demonstrate not only efficacy relative to placebos, but clinical superiority compared to existing drugs, while Hollis [2004] offers a similar but more tempered version of the proposal.^9^ On the other hand, DiMasi and Paquette [2004] argue that me-too's provide therapeutic options previously not available, and that me-too's are often engaged in development concurrently with the pioneering drug. Thus, changes in the FDA approval policy would create moving targets in the clinical trial phase since the developers would have to account for the possibility of being second to reach the market and create tests to show superiority over the winning first developer. Note that all four introductions that we focus on are me-too drugs: none were the first drug in the therapeutic class, each filed an application with the FDA for a new formulation (rather than a new chemical entity) and each received a standard review rating from the FDA (rather than a priority review). Yet two drugs (Adderall XR and Concerta) generated welfare gains which were larger than those of the generic introduction of MAS-IR while the other two (Metadate CD and Methylin ER) resulted in gains that were about an order of magnitude smaller. As our results suggest, not all me-too's are created equal and over-arching proposals aimed at slowing the introduction of all me-too's may not be appropriate.

Our results also provide a *rough estimate* of a potential welfare loss due to entry that did not take place. Shire holds two key patents on Adderall XR that technically prevent entry in the MAS-ER segment until 2018 and an exclusivity period until April, 2005, under the Hatch-Waxman Act.^10^ However, Barr Laboratories filed for an ANDA application with the FDA in February, 2003, to market a generic version of Adderall XR (Barr Laboratories, Inc. []2003c). This was followed by a second ANDA application filed by IMPAX in November, 2003, (Impax Laboratories, Inc. [2003]). In response, Shire sued Barr as well as IMPAX for infringement of its key patents (Barr Laboratories, Inc., [, a2003b]).^11^ The case between Barr and Shire was scheduled to go to trial in January, 2006, and would have granted Barr 180 days of generic exclusivity under section IV of the Hatch-Waxman Act *if* it won the case while IMPAX, as a second filer of an ANDA, would not have gained an exclusivity period. However, in the same month (January, 2006), Shire settled with IMPAX, the second filer of ANDA, to market Adderall XR under a license from Shire *no later than* January, 2010 (FDAnews Drug Daily Bulletin [2006]). This deal was followed by a second out of court settlement (August, 2006), this time between Shire and Barr, the original filer of ANDA, where Shire agreed to grant Barr Laboratories a 180-day *exclusive* license to market generic Adderall XR in exchange for delaying entry until April, 2009 (Barr Laboratories. [2006], Patel, [2006]).^12^

The out of court settlements between Shire, Barr and IMPAX bear features noted in several recent cases where, in exchange for delayed entry, the agreement includes a ‘reverse payment’ from the patent holder to the generic maker but allows for generic entry prior to patent expiration (see Bulow [2004], Hemphill [2007], Frank [2007]).^13^ Like some of the earlier similar cases where the FTC contested the settlements, the FTC initiated an initial inquiry in October, 2006, and in June, 2007, Shire received a civil investigative demand from FTC relating to its settlement with Barr and its earlier settlement with IMPAX. These settlements also highlight loopholes in the Hatch-Waxman Act [FTC, 2011]. For instance, while the Act provides a 180-day exclusivity period to the first filer of ANDA (to give incentives for generic entry), it does not prevent the original patent holder from licensing its drug to another generic maker which in effect nullifies the 180-day exclusivity of the first generic entrant. Note that the drug that did not enter (Barr's generic Adderall XR) shares attributes with two other drugs for which welfare effects have been estimated: it is like the generic MAS-IR (welfare effect $65-$43 per child per year) since it is a generic in the same molecule class, and it is also like the branded Adderall XR (welfare effect $123-$119 per child per year) since it is a 12-hour drug in the same molecule class. While it is difficult to predict the outcomes of the court proceedings and when first generic entry would have taken place in the absence of any out of court settlements between these firms, nonetheless, our estimates suggest that even a year earlier entry in generic MAS-ER segment (Barr's generic Adderall XR) could have been at least equal to the lower of the two welfare effects estimated above.

## Footnotes

Me-too drugs are not generics. They are patented ‘follow-on’ drugs, meaning they are are not the first ones within their class but a patent has been granted presumably because they contain unique variants of the original drug in the class.

CIBA Pharmaceutical merged with J. R. Geigy, Ltd., to form Ciba-Geigy in 1970. Ciba-Geigy merged with Sandoz Laboratories in 1996 to form Novartis, which is now the producer of Ritalin.

For instance, both Ritalin LA and Metadate CD use a bead-delivery system where the active molecule (methylphenidate) is packed into two types of beads, rapid-release which reaches the bloodstream quickly and extended-release beads which dissolve slowly. The primary difference is that Metadate CD uses 30% of rapid-release beads while Ritalin LA uses 50% of rapid-release beads leading to a difference in the absorption profile across the two drugs. By contrast, Concerta uses a membrane based technology called Osmotic Release Oral System (OROS). The tablet is coated with methylphenidate which dissolves immediately into the blood and exposes a membrane with multiple layers of the drug. As water seeps slowly through the membrane, it pushes additional drug out to the body and the thickness of the membrane determines the delivery time.

The grouping in the table is similar to one in Conner [2006]. However, the dosage equivalence was compiled by Professor Steve Hinshaw (UC Berkeley) and Dr. Peter Levine (pediatrician with Kaiser Permanente of Northern California). We are indebted to Professor Hinshaw and Dr. Levine for providing us with the equivalence table.

Methylin ER is a generic extended release MPH version of Ritalin SR but is sold under a trade name since the immediate release version Methylin (also by Mallinckrodt) was already sold as a branded drug—an NDA application was filed for Methylin with the FDA but an ANDA application was filed for Methylin ER. A similar issue applies to Metadate ER, also a generic drug (with an ANDA application) but sold under the trade name of Metadate ER.

Unlike the earlier 8-hour release tablets, Metadate CD is a capsule with biphasic release, meaning there is an initial rapid release of methylphenidate, followed by a continuous-release phase (by contrast, for instance, the Metadate ER tablet dissolves slowly over the eight hours as it passes through the gastrointestinal tract).

The primary difference is that Metadate CD releases 30% of the drug initially and the remaining 70% over an extended period of time while Ritalin LA has an initial rapid release of 50%.

For instance, Cylert (pemoline) specifically mentions on the label that it should not be considered as a first line therapy for ADHD, and comes with the requirement that the prescribing physician obtain written consent from the patient prior to prescribing this drug. Similarly, while Modafinil is approved by the FDA for narcolepsy and a few other uses, it is not FDA-approved for ADHD. However, some physicians do prescribe it for ADHD. Thus due to the unique nature of each of these drugs, we kept them in a separate segment.

Proponents offer several arguments such as: (a) me-too's are similar to the pioneering drugs and the incremental benefits and benefit/risk ratios are low; (b) me-too's split the market as opposed to expanding it, shorten the exclusivity period for the pioneering drugs, and reduce the incentives to undertake R&D and, (c) pharmaceutical companies expend significant resources on marketing their me-too drugs rather than on R&D itself (Hollis [2004]).

There was originally only one patent listed in the Orange Book and the original exclusivity period was until October, 2004, but a six month extension was later granted for Adolescent Pediatric Patients.

Barr Laboratories maintained that the patents listed by Shire are invalid, unenforceable and/or will not be infringed by Barr Laboratories.

On April 2, 2009, Teva Pharmaceuticals (which now owns Barr) commenced shipping generic Adderall XR in the U.S and IMPAX started shipping a generic version of Adderall XR on October 2, 2009 (Teva Pharmaceutical Industries Ltd [2009], Impax Laboratories, Inc. [2009]).

Shire noted in its press release that no payments had been made to Barr in settlement of the Adderall XR dispute. Nonetheless, the ‘reverse payments’ existed as a result of complex side deals. The deal involved a payment from Shire to Duramed, a subsidiary of Barr in the amount of $165 million described as compensation for product development related to transvaginal ring technology for urinary incontinence, which Shire planned to apply to five women's health products, and an oral contraceptive called Seasonique. In addition, Shire sold the rights to its older Adderall immediate release (IR) product to Duramed for $63 million, resulting in a net payment of $102 million by Shire (see Barr Laboratories, Inc.[2006]).

## APPENDIX A

### Estimation Details

#### Dosage Equivalence

When a patient switches from one drug to another drug, the conversion is not always gram for gram. Thus, in the demand estimation, we converted the prices from dollars per gram to dollars per defined monthly dosage (DMD) using the dosage equivalence given in Table I and World Health Organization's (WHO) definition for defined daily dosage (DDD). For the four hour MPH (i.e., Ritalin), WHO defines 30mg as DDD or 0.9 grams per month. Thus, if the price per gram of Ritalin is *p_r_*, then the price per month is 30 × 30 × *p_r_*/1000 = 0.9 × *p_r_*. For other drugs (not all are listed in the WHO database), we first apply a dosage equivalence and then multiply by 0.9. For example, the price of Concerta per gram in 2003 is $73.94, but 1mg of Concerta is equivalent to 0.69mg of Ritalin and hence the price per month of Concerta is $73.94 × 0.9/0.69 = $96.45. Thus, we are assuming that a typical patient consumes 0.9 grams of Ritalin per month and if they are on another drug, appropriate dosage conversion is used for grams per month. The robustness section reports differences in estimates without these conversions. The table below is similar to the one in the main text (Table II) except that it provides prices in dollars per Ritalin equivalent grams per month.

**Table AI:** Average Prices and Shares by Year (778 MSA Counties)

		Price per Defined Monthly Dosage (DMD) /Std					Share of Revenue				
		1999	2000	2001	2002	2003	1999	2000	2001	2002	2003
(1)	Ritalin (1.0)	48.6/3.5	47.99/3.5	47.53/3.9	48.25/4.5	52.41/5.9	0.117	0.081	0.033	0.016	0.009
(2)	Methylin (1.0)	40.74/5.4	37.68/5	35.96/5.9	34.12/6	31.81/6.3	0.045	0.068	0.035	0.022	0.013
(3)	Gen MPH-IR (1.0)	39.32/2.7	37.4/3.7	35.77/3.9	34.72/4	33.06/4.1	0.289	0.185	0.098	0.049	0.026
(4a)	Ritalin SR (0.83)	66.43/2.3	66.56/2.3	67.6/2.6	69.83/3.5	76.44/4	0.047	0.032	0.012	0.006	0.003
(4b)	Ritalin LA (1.25)	.	.	.	56.94/6.3	57.25/4.6	.	.	.	0.006	0.024
(5a)	Metadate CD (1.25)	.	.	38.38/1.6	43.06/1.7	56.22/2.4	.	.	0.006	0.025	0.024
(5b)	Metadate ER (0.83)	NA	65.33/13.4	64.28/10.3	64.75/10.5	66.11/12	NA	0.007	0.007	0.005	0.002
(6)	Methylin ER (0.83)	.	58.41/10.1	56.62/9.5	52.6/7.9	52.29/7.7	.	0.004	0.010	0.008	0.006
(7)	Gen MPH-ER (0.83)	54.21/2.7	52.78/3.5	50.28/5.4	49.96/5.5	48.12/6.2	0.105	0.080	0.034	0.017	0.008
(8)	Concerta (0.69)	.	110.33/9.9	92.96/7.5	90.82/6	96.45/5.6	.	0.047	0.238	0.298	0.261
(9)	Adderall (2.86)	17.79/0.8	19.86/0.9	29.48/2.8	32/3.9	31.87/5.3	0.216	0.311	0.358	0.114	0.029
(10)	Gen MAS-IR (2.86)	.	.	.	29.02/4.2	26.52/4.1	.	.	.	0.084	0.076
(11)	Adderall XR (2.14)	.	.	47.44/6.6	49.14/3.9	52.58/3.7	.	.	0.011	0.202	0.238
(12)	Dexedrine (1.75)	25.55/1	27.36/1.1	30.41/1.4	34.03/1.8	34.94/1.9	0.013	0.010	0.006	0.003	0.002
(13)	Dextrostat (1.75)	21.63/1.2	23.59/1.4	28.02/1	28.79/2.1	23.25/2.2	0.016	0.018	0.012	0.005	0.002
(14)	Gen DEX-IR (1.75)	.	.	26.31/2.3	25.27/2.5	24.62/2.1	.	.	0.003	0.004	0.004
(15)	Dexedrine SR (2.14)	28.45/1.8	32.08/2	35.84/2.2	39.6/2.9	40.25/4.1	0.062	0.062	0.045	0.022	0.007
(16)	Gen DEX-ER (2.14)	.	.	.	35.59/3	35.14/3.3	.	.	.	0.009	0.011
(17a)	Cylert (0.44)	81.05/4.4	85.89/6	87.14/7.7	90.59/9.4	90.06/9.1	0.061	0.023	0.009	0.004	0.002
(17b)	Provigil(0.28)	80.04/7.3	79.13/5.3	83.93/5.6	86.47/5.3	89.85/5.2	0.022	0.058	0.072	0.093	0.094
(17c)	Gen Pemoline (0.44)	66.41/8.2	64.76/8	67.68/9.3	64.36/11	60.49/13.4	0.005	0.015	0.011	0.006	0.004
(17d)	Strattera (0.83)	.	.	.	.	83.59/6.4	.	.	.	0.000	0.156
Total Revenue (in Millions)							688.5	836.8	1,110.3	1,438.0	1,992.7

*Note 1:* Prices are in constant 2000 dollars. Missing value implies the drug was not on the market, except for the case of Metadate ER which was on the market in 1999, but data are not available to us.

*Note 2:* Total revenue (also in constant 2000 dollars) is only for the drugs listed above from the 778 counties and does not include mail order sales.

*Note 3:* The number in parenthesis is the conversion factor used for converting to generic MPH-IR equivalent dosage prices. Example: the price of Concerta per gram in 2003 is $73.94, but 1mg of Concerta is equivalent to 0.69mg of Ritalin and hence the dosage adjusted price of Concerta is $73.94/0.69 = $107.17. This multiplied by 0.9 can be read as ‘price per defined monthly dosage’ of Ritalin. To obtain price per gram, multiply by the conversion factor and divide by 0.9.

#### Fixed Shares

To avoid the obvious endogeneity problem that is introduced in share equations (1a, (1b)) due to the use of the Stone-index (since then the share appears on both the left and right hand side of the estimated equation, see equation (2)), we follow Hausman and Leonard [2002] and construct an alternative Stone-index using  which is the average share of the drug *i*, *f*, *m* over all areas at time period *t*. This alternative Stone-index is only used during estimation of the segment equations. Hausman and Leonard [2002] in fact constructed the alternative Stone index from area-specific shares averaged over the full sample period. However, the imbalance in the number of years drugs are present in our data, and the fact that our panel is quite short to begin with, argues against the construction of area specific time-averaged share measures for the Stone indexes. Thus we use year specific area-averaged share measures across all counties in the data. Specifically, we use . Further, our results are not sensitive to this choice.

#### Segment by Segment estimation

Note that there are five sets of bottom level share equations, one for each of the segments (1) MPH-IR (with three share equations), (2) MPH-ER (with four share equations), (3) MAS-IR (with two share equations), (4) DEX-IR (with three share equations) and, (4) DEX-ER (with two share equations). At level 2, three mid-level segments need to be estimated. These are (1) MPH, where the shares are of IR, ER and OROS (i.e., Concerta), (2) MAS, where the shares are of IR and ER (i.e., Adderall XR) and (3) DEX, where the shares are of ER and IR. At the next level up, there are four quantity equations and at the top level there is only one equation.

The segment estimations are carried out separately and independently rather than in a full joint system estimation because not all drugs are available in the market throughout the study period. For instance, the bottom level segments, MPH-IR and MPH-ER are observed for years 2000 to 2003 and hence these segments can be estimated on a panel of 4 years of data. However the drugs in the bottom level segment MAS-IR are observed for only two years (see Table II). While using a full joint estimation across all segments provides a potential gain in efficiency—by using cross-equation correlations across segments—it requires finding a common set of years where all drugs are present. But this results in a substantial loss of data since now all segments can only by estimated for two years of data rather than some that can be estimated using four years of data. Note however that within each segment, equations are still estimated jointly as a system. In the latter part of the paper we compute and provide estimates of unconditional elasticities which require values of parameters from different segments. Since each segment is estimated (as a system) separately, covariances among parameters in different segments are not available. Hence, use of delta methods to construct standard errors of unconditional elasticities are invalid and hence we used bootstrap methods to construct confidence intervals.

#### Welfare computation

Two adjustments were made in the computations of NB equilibrium prices. First, the computed marginal cost of Methylin was negative (due to inelastic estimated demand). Consequently, we set it's elasticity equal to that of the generic in the segment (which is almost equivalent to setting its marginal cost equal to that of the generic). Note, if we left the marginal cost of Methylin at its original negative value, the overall welfare effects were quite similar. Second, as noted in the paper, the demand equation for drug 17 (an aggregate of four different drugs), was sensitive to small changes in the model specification. The point estimates of the cross price elasticities of this drug with respect to the remaining 16 would change with small changes in the model. In turn, the estimates of the NB equilibrium prices (in the but-for world) and the corresponding CV calculations would also change. To overcome this difficulty, we set the off-diagonal in the elasticity matrix associated with drug 17 to zero. This was so that when the Nash-Bertrand equilibrium prices are computed for the remaining 16 drugs in the but-for world, the cross-effects with drug 17 would not impact those calculations. Thus, in the but-for world calculations, the price of drug 17 does not change nor does it interact with the remaining 16 drugs.

#### Supplemental Materials

The Journal's web site provides supplemental materials referenced in the article. These include (a) restriction tests (homotheticity, homogeneity and symmetry), and (b) IV regression coefficients for all segments.

#### Derivation of Elasticities

This section derives the conditional and unconditional elasticities (a more condensed version for a two-level system is given in Ellison, Cockburn, Griliches *et* *al*. [1997]). Observe that  and hence . Thus, elasticity of *ith* drug in f-m segment with resect to *kth* drug in *f*′ − *m*′ segment is given by

A-1

where 1[.] is an indicator function equal to 1 when the statement inside is true and is otherwise 0. Note that the subscripts are such that if *m* ≠ *m*′ then it automatically implies that *f* ≠ *f*′ and *i* ≠ *k*. Similarly, if *f* ≠ *f*′ then *i* ≠ *k*.

#### Elasticities Conditional on Segment Revenue

We first compute elasticities conditional on the revenue segment . Then in equation ((A-1)) we can set the partial of  equal to zero. From equation (1a) we have

where once again, in equation (1a) we have set the partial of  equal to zero. Next, from equation (2a), the partial of the fixed-weight Stone index of segment f-m with respect to  is zero if *m*′ ≠ *m* and *f*′ ≠ *f* but otherwise is . Hence the elasticity (conditional on  ) is

A-2

Thus, elasticities conditional on  are zero across drugs in different f-m segments. Incidently, observe that by the same logic, elasticities of forms within a molecule with respect to the *price index* for the form, conditional on *R_m_*, has a similar formula and is given by

A-3

#### Unconditional Elasticities

Share of drug *i* in segment f-m is affected by the price of drug *k* in some other segment f′-m′ only via the change in the expenditures of the segments. Specifically, when the price of drug *k* in segment f′-m′ changes, it changes the price index of segment f′-m′. When the price index of segment f′-m′ changes, it changes the relative prices of segments f′-m′ and f-m. This in turn changes the share of revenue spent on segment f-m vs. f′-m′ and is determined by the middle level share equations. Thus when computing the relevant partials we do not hold  (or *R_m_*) constant. Further, the link between the bottom and middle level equations is established by considering the ratio  in equation (1a) as a measure of a standard unit of quantity of molecule m and form f, i.e.  and since  , we substitute  for  in the bottom level share equation (1a). Similarly, in the middle level share equation (1b), we substitute *Q_m_* from the top level quantity equation (1c).

The general expression for elasticities is given in equation ((A-1)). We evaluate each of the partials above while making the appropriate substitutions. First, observe that by substituting  for  in the bottom level share equation (1a) we get  and hence

Similarly, since  hence

Substituting these two partials in the general elasticity equation ((A-1)) above we get,

A-4

Thus, we need to evaluate  and substitute it in the equation above. Note that  and hence

A-5

To evaluate the two partials in ((A-5)) above, we substitute *Q_m_* for *R_m_*/*P_m_* in the middle level share equation (1b), i.e.,  and use the chain rule. Thus,

A-6

Hence equation ((A-5)) becomes

A-7

We can substitute equation ((A-7)) back into ((A-4)) to get elasticities in terms of these new partials as

A-8

Note that the remaining partials are with respect to the (log of) price indexes  as opposed to with respect to the (log of) actual prices and are easily evaluated from the middle and top level equations. Thus from the middle level equation  (with *Q_m_* substituted in for *R_m_*/*P_m_*) we get,

A-9

Next, since  therefore  but  and hence

A-10

Finally, from the top level quantity equation  we get

A-11

where note that in this last partial derivative there is no indicator function since the top level equation itself consists of all molecule level price indexes.

Thus, we can substitute equations ((A-9), (A-10) and (A-11)) into ((A-8)) to get the final expression for elasticities in terms of the shares and model parameters. Upon substitution this gives the unconditional elasticities as

A-12

**Table AII:** Unconditional Elasticities from IV (3SLS) Estimates

**Table AIII:** Unconditional Elasticities from OLS (SUR) Estimates

## APPENDIX B

This appendix provides three sets of results mentioned in the paper: (a) restriction tests, (b) full unconditional elasticity matrices (SUR and 3SLS) and, (c) regression coefficients.

### Restriction Tests

**Table BI:** Homogeneity and Symmetry Restrictions Tests

	ChiSq	DF	p-value
MPH-IR	17.01	3	0.0007
MPH-ER	10.63	6	0.1005
MPH	8.364	3	0.0391
MAS-IR	3.080	1	0.0792
MAS	21.92	1	0.0000
DEX-IR	0.411	3	0.9379
DEX-ER	0.224	1	0.6359
DEX	0.187	1	0.6653
Molecules	20.62	10	0.0239
Molecules (MPH, MAS, DEX Symmetry only)	1.258	3	0.7391

*Note*: The chi-square test statistic is the Wald statistic computed as  where  is the column vector of restrictions,  ,  are the unrestricted estimated coefficients and  is the variance-covariance matrix of the unrestricted coefficients. The DF column provides the number of restrictions being jointly tested. Thus, for instance, in the MPH-IR segment, since there are three share equations, 2 homogeneity and one symmetry restriction is tested (the parameters of the last equation are not tested since conservation, i.e., shares, add-up to one, is still imposed in these tests). The last test is when only symmetry is imposed between MPH, MAS and DEX.

**Table BII:** Homotheticity Tests

	ChiSq	DF	p-value
MPH-IR	21.45	2	0.0000
MPH-ER	268.5	3	0.0000
MPH	150.4	2	0.0000
MAS-IR	10.29	1	0.0013
MAS	63.53	1	0.0000
DEX-IR	15.01	2	0.0005
DEX-ER	0.202	1	0.6534
DEX	55.69	1	0.0000
Molecules	24.46	4	0.0001

*Note*: The chi-square test statistic is the usual Wald statistic. The tests given above are joint tests for homothecity within a segment, i.e., the coefficients on ln(R/P) are each different from zero. However, in the share equations, homogeneity and symmetry are already imposed. Also, since the shares must add to one, the last share equation within each segment is never estimated. Thus, in a segment with 3 share equations, the joint test is that *β*_1_ = 0 and *β*_2_ = 0 (but *β*_3_ = 0 cannot be tested). Similarly, in segments where there are just two share equations, the joint test is just a single test, i.e., *β*_1_ = 0 and in these cases, the chi-sq statistic is just the square of the z-statistic on the regression coefficient. In the molecules segment (m3) homogeneity is not imposed (also since these are not shares equations, conservation is also not imposed) and symmetry is imposed within the MPH, MAS and DEX molecules.

#### Regression Coefficients

The following applies to all regression results reported below. (1) Each segment is estimated separately as a system (3SLS). The last equation in each segment is not estimated but is implied by system restrictions (i.e., homogeneity, symmetry and conservation were imposed). (2) All regressions include state dummies but are not displayed. (3) The table shows the mean and standard deviations of the bootstrap regression coefficients. (4) The numbers 1, 2*, …* on log prices refer to the price of drugs 1, 2*, …* in the segment (i.e., are re-set to 1 within each segment). Similarly, *ln*(*R/P* ) refers to the coefficient on log of ratio of expenditure to price index for the segment. The exception is the molecule level segment (level 3 Cobb-Douglas equations) where homogeneity is not imposed and symmetry restrictions are imposed only between MPH, MAS and DEX.

**Table BIII:** Regression Coefficients for Segments MPH-IR, MPH-ER and MPH

	MPH-IR (M1F1)			MPH-ER (M1F2)				MPH (M1)		
	s1 (Ritalin)	s2 (Methylin)	s3 (Generics)	s1 (Ritalin SR/LA)	s2 (Metadate ER/CD)	s3 (Methylin ER)	s4 (Generics)	u1 (MPH-IR)	u2 (MPH-ER)	u3 (MPH-OROS) (Concerta)
ln(R/P)	0.012	0.006	−0.018	0.002	0.051	−0.008	−0.045	−0.024	−0.001	0.025
	0.004	0.003	0.004	0.005	0.003	0.002	0.005	0.002	0.002	0.003
lnp1	−0.226	0.059	0.167	−0.535	0.081	0.267	0.187	−0.334	−0.17	0.504
	0.086	0.059	0.064	0.173	0.072	0.067	0.174	0.117	0.031	0.099
lnp2	0.059	0.146	−0.205	0.081	−0.235	0.005	0.148	−0.17	−0.243	0.413
	0.059	0.065	0.06	0.072	0.095	0.047	0.077	0.031	0.014	0.034
lnp3	0.167	−0.205	0.038	0.267	0.005	−0.214	−0.058	0.504	0.413	−0.917
	0.064	0.06	0.078	0.067	0.047	0.056	0.082	0.099	0.034	0.095
lnp4				0.187	0.148	−0.058	−0.278			
				0.174	0.077	0.082	0.222			
time	−0.099	−0.414	0.513	0.527	−1.51	0.812	0.173	−0.434	−0.143	0.577
	0.01	0.022	0.023	0.191	0.085	0.089	0.238	0.042	0.012	0.037
timeSq	0.014	0.135	−0.149	−0.214	0.525	−0.21	−0.101	0.039	0.019	−0.058
	0.002	0.007	0.007	0.055	0.032	0.028	0.072	0.006	0.002	0.005
timeCb		−0.013	0.013	0.026	−0.053	0.017	0.009			
		0.001	0.001	0.005	0.004	0.003	0.007			
lnkids	−0.007	−0.015	0.022	−0.008	−0.017	0.016	0.009	0.014	0.001	−0.016
	0.005	0.004	0.005	0.006	0.005	0.003	0.007	0.003	0.002	0.004
lnmds	0.018	−0.011	−0.007	0.022	−0.026	0.002	0.002	0.012	−0.001	−0.011
	0.003	0.003	0.003	0.003	0.003	0.002	0.003	0.002	0.002	0.002
lncaid	0.011	−0.007	−0.004	0.027	−0.026	−0.01	0.009	−0.001	0.012	−0.011
drugs	0.003	0.004	0.004	0.006	0.005	0.003	0.005	0.003	0.003	0.004
lncaid	−0.004	−0.031	0.035	0.008	−0.009	−0.001	0.002	−0.084	−0.001	0.085
enrollees	0.009	0.009	0.011	0.014	0.014	0.01	0.015	0.006	0.007	0.009

**Table BIV:** Regression Coefficients for Segments MAS-IR and MAS

	MAS-IR (M2F1)		MAS (M2)	
	s1 (Adderall)	s2 (Generics)	u1 (MAS-IR)	u2 (MAS-ER Adderall XR)
ln(R/P)	−0.017	0.017	−0.029	0.029
	0.005	0.005	0.004	0.004
lnp1	−0.568	0.568	−0.164	0.164
	0.349	0.349	0.106	0.106
lnp2	0.568	−0.568	0.164	−0.164
	0.349	0.349	0.106	0.106
time	−0.263	0.263	−1.350	1.350
	0.027	0.027	0.061	0.061
timeSq			0.126	−0.126
			0.009	0.009
lnkids	−0.003	0.003	0.009	−0.009
	0.006	0.006	0.003	0.003
lnmds	0.027	−0.027	0.015	−0.015
	0.004	0.004	0.002	0.002
lncaid	0.003	−0.003	−0.000	0.000
drugs	0.005	0.005	0.003	0.003
lncaid	0.026	−0.026	−0.035	0.035
enrollees	0.024	0.024	0.010	0.010

**Table BV:** Regression Coefficients for Segments DEX-IR, DEX-ER and DEX

	DEX-IR (M3F1)			DEX-ER (M3F2)		DEX (M3)	
	s1 (Dexedrine)	s2 (Dextrostat)	s3 (Generics)	s1 (Dexedrine SR)	s2 (Generics)	u1 (DEX-IR)	u2 (DEX-ER)
ln(R/P)	−0.017	0.011	0.006	−0.003	0.003	−0.027	0.027
	0.004	0.004	0.003	0.007	0.007	0.004	0.004
lnp1	−0.908	0.629	0.278	−0.38	0.38	0.012	−0.012
	0.504	0.319	0.34	0.592	0.592	0.087	0.087
lnp2	0.629	−0.706	0.076	0.380	−0.380	−0.012	0.012
	0.319	0.291	0.219	0.592	0.592	0.087	0.087
lnp3	0.278	0.076	−0.355				
	0.340	0.219	0.330				
time	−0.214	0.169	0.044	−0.304	0.304	0.004	−0.004
	0.213	0.200	0.223	0.017	0.017	0.011	0.011
timeSq	0.038	−0.055	0.023			−0.002	0.002
	0.028	0.028	0.006			0.001	0.001
lnkids	−0.018	−0.005	0.023	−0.008	0.008	−0.006	0.006
	0.008	0.007	0.006	0.010	0.010	0.005	0.005
lnmds	0.044	−0.018	−0.025	0.022	−0.022	0.027	−0.027
	0.006	0.005	0.004	0.005	0.005	0.004	0.004
lncaid	0.008	−0.009	0.001	−0.011	0.011	−0.001	0.001
drugs	0.008	0.008	0.008	0.007	0.007	0.004	0.004
lncaid	0.001	−0.036	0.036	−0.030	0.030	−0.018	0.018
enrollees	0.021	0.02	0.021	0.039	0.039	0.009	0.009

**Table BVI:** Regression Coefficients for Levels 3 and 4 Equations

	Level 3				Top Level	
	lnQ1 (MPH)	lnQ2 (MAS)	lnQ3 (DEX)	lnQ4 (OTH)		lnQ (All)
lnR	0.955	1.077	1.046	0.935	lnY	0.213
	0.012	0.018	0.029	0.065		0.057
lnp1	−1.20	0.765	0.272	2.178	lnP	−1.210
	0.413	0.384	0.703	2.168		0.420
lnp2	0.765	−1.490	1.488	−4.21		
	0.384	0.564	0.724	2.17		
lnp3	0.272	1.488	−1.690	−0.39		
	0.703	0.724	1.42	2.898		
lnp4	0.782	−1.23	−2.190	−1.25		
	0.359	0.480	1.080	1.914		
s8	0.605	−0.711	−0.39	−2.38		
	0.320	0.303	0.536	1.67		
s11	−0.649	0.586	−1.78	2.487		
	0.218	0.318	0.416	1.237		
time	−4.36	2.597	−8.68	17.47	time	0.243
	1.199	1.775	2.270	6.691		0.141
timeSq	1.130	−0.632	2.501	−4.68	timeSq	0.011
	0.301	0.446	0.585	1.717		0.010
timeCb	−0.097	0.048	−0.234	0.419		
	0.025	0.037	0.050	0.144		
lnkids	0.027	−0.02	−0.074	−0.034	lnkids	0.384
	0.010	0.017	0.030	0.043		0.056
lnmds	−0.002	−0.05	0.076	0.187	lnmds	0.496
	0.006	0.009	0.019	0.029		0.025
lncaid	−0.017	−0.028	−0.043	0.011	lncaid	0.034
drugs	0.009	0.01	0.023	0.036	drugs	0.012
lncaid	0.107	−0.167	−0.005	−0.128	lncaid	0.026
enrollees	0.025	0.031	0.052	0.129	enrollees	0.042
